# PRMT9 Aggravated Dopaminergic Neurodegeneration in Parkinson's Disease Model by Facilitating the Degradation of DUSP26 and Inducing Mitochondrial Dysfunction

**DOI:** 10.1002/advs.76033

**Published:** 2026-06-09

**Authors:** Tengfei Liu, Xiaomeng Song, Xin Guo, Rui Xiao, Ru Sun, Qiuran Ji, Lu Yu, Yiquan Li, Qingyi Fu, Qidi Xue, Lingjun Kong, Lin Chen, Chengjiang Gao, Huiqing Liu

**Affiliations:** ^1^ Department of Pharmacology School of Basic Medical Sciences and Qilu Hospital Cheeloo College of Medicine Shandong University Jinan Shandong P. R. China; ^2^ Research Center for Basic Medical Sciences Qilu Hospital Shandong University Jinan Shandong P. R. China; ^3^ Department of Neurosurgery The First Medical Centre Chinese PLA General Hospital Beijing P. R. China; ^4^ Department of Pharmacy Shandong Provincial Hospital Affiliated to Shandong First Medical University Jinan Shandong P. R. China; ^5^ Key Laboratory of Infection and Immunity of Shandong Province & Department of Immunology School of Basic Medical Sciences Shandong University Jinan Shandong P. R. China; ^6^ Shenzhen Research Institute of Shandong University Shenzhen Guangdong P. R. China

**Keywords:** arginine methylation, DUSP26, mitochondria, neurodegeneration, Parkinson's disease, PRMT9

## Abstract

Protein arginine methyltransferases (PRMTs) catalyze protein arginine methylation and play a crucial role in the pathogenesis of diseases. Mitochondrial dysfunction is implicated in the neurodegeneration of Parkinson's disease (PD). However, whether protein arginine methyltransferase 9 (PRMT9), which is found in mitochondria, is involved in PD remains unclear. In this study, we found that PRMT9 was markedly increased in the nigrostriatal region of PD mice induced by MPTP and in SH‐SY5Y cells stimulated with MPP^+^. In MPP^+^‐exposed SH‐SY5Y cells, PRMT9 translocated to mitochondria and exacerbated mitochondrial injury, manifested as decreased mitochondrial membrane potential, reduced ATP production, elevated ROS levels, and mitochondrial fragmentation. Furthermore, PRMT9 deficiency significantly alleviated dopaminergic (DA) neurodegeneration induced by MPTP, while PRMT9 overexpression aggravated MPTP‐induced neurodegeneration. Mechanistically, PRMT9 directly interacted with dual‐specificity phosphatase 26 (DUSP26) and catalyzed its arginine methylation at residue R29, which then promoted the polyubiquitination and proteasomal degradation of DUSP26 mediated by Trim32. Therefore, PRMT9 drove DA neurodegeneration in PD by promoting DUSP26 degradation and inducing mitochondrial dysfunction. Our study identified PRMT9 as a novel potential therapeutic target and a pharmaceutical intervention targeting the interaction between PRMT9 and DUSP26 may provide a promising strategy for PD.

## Graphical abstract

In the pathological state of PD induced by MPP^+^, the upregulated PRMT9 in dopaminergic neurons translocates into mitochondrion and interacts with DUSP26 and catalyzes its arginine methylation, leading to the ubiquitin‐proteasomal degradation of DUSP26 mediated by Trim32. Thus, the low protein level of DUSP26 induces higher ROS production and the collapse of the mitochondrial membrane potential, leading to mitochondrial dysfunction and subsequent neurodegeneration, which finally promotes PD progression.

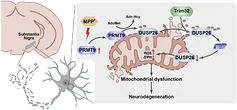

## 1 Introduction

Parkinson's disease (PD) is the second most common neurodegenerative disorder [1, 2]. The progressive loss of dopaminergic (DA) neurons in substantia nigra pars compacta (SNpc) causes the motor abnormalities characterized as tremor, rigidity and abnormal gait [3, 4, 5]. Conventional treatments for PD alleviate symptoms but fail to halt neurodegeneration [6], due to the fact that the pathogenesis of PD has not been fully elucidated.

Arginine methylation catalyzed by protein arginine methyltransferases (PRMTs) is a critical post‐translational modification (PTM). Accumulating studies have shown that arginine methylation was closely related to neurodegenerative diseases by regulating signal transduction, RNA splicing and protein stability [7, 8, 9]. PRMT1‐mediated methylation of FUS modulated its nucleocytoplasmic shuttling, a process linked to FUS aggregation in amyotrophic lateral sclerosis (ALS) [10]. In PD, α‐synuclein (α‐syn) bound to the BAF complex and recruited PRMT5, thereby promoting symmetric dimethylation (SDMA) of histone H4 at arginine 3 (H4R3me2s) and repressing the transcription of neuronal differentiation‐related genes, ultimately exacerbating DA neuronal impairment [11]. Of note, mitochondrial dysfunction constitutes a core pathological feature in both sporadic and familial PD [12, 13, 14]. PRMT9, one of SDMA‐forming PRMTs, is characterized by a unique duplicated methyltransferase domain. PRMT9, which was found in mitochondria, targeted to the mitochondrial protein MAVS and mediated its arginine methylation, then restrained the activation of MAVS to preserve innate immune homeostasis [15]. In addition, PRMT9 was identified as a potential therapeutic target in acute leukemia [16]. More recently, PRMT9 was found to be catalytically inactive in intellectual disability patients and function in synaptic plasticity [17]. Public GEO transcriptomic data GSE168496 further revealed the mRNA expression of PRMT9 was significantly elevated in the SNpc of PD patients [18]. Nevertheless, the specific functional link between PRMT9 and PD remains poorly understood.

Dual‐specificity phosphatase 26 (DUSP26) is located in the mitochondrial outer membrane (OMM) and belongs to the dual specificity phosphatase family. Traditionally, DUSP26 has been shown to regulate p38 and ERK1/2 activities during cell proliferation, differentiation and death [19]. In addition, DUSP26 acts as a p53 phosphatase in neuroblastoma cells, and its inhibition triggers p53‐mediated apoptosis [20]. Recently, it was reported that DUSP26 influenced mitochondrial function through the FAK‐ERK signaling axis [21] and played a pivotal role in axonal regeneration and functional recovery [22]. Of note, DUSP26 was highly expressed in neurons of different brain regions, including the cortex and midbrain, and displayed a significant reduction in the SNpc of PD patients [23]. Moreover, DUSP26 ablation induced a significant impairment in mitochondrial function and resulted in DA neuron loss in the SNpc of mice [23]. However, few studies about the PTM of DUSP26 were carried out.

Given that PRMT9 levels rose while DUSP26 levels fell in PD models, we hypothesized that PRMT9 might be a direct upstream negative regulator of DUSP26 stability. In this study, we found that PRMT9 was markedly increased both in the nigrostriatal region of PD mice induced by MPTP and in SH‐SY5Y cells stimulated by MPP^+^. Moreover, PRMT9 obviously translocated into mitochondria and further aggravated mitochondrial dysfunction and neurodegeneration induced by MPP^+^ through regulating DUSP26 stability. Consistently, PRMT9 deficiency significantly alleviated DA neurotoxicity induced by MPTP. Mechanistically, MPP^+^ stimulation enhanced the interaction between PRMT9 and DUSP26. PRMT9 subsequently catalyzed DUSP26 arginine methylation at R29, which then promoted its polyubiquitination and proteasomal degradation mediated by E3 ubiquitin ligase Trim32. Thus, PRMT9 would be a potential therapeutic target for the treatment of PD.

## 2 Results

### 2.1 PRMT9 Aggravated MPP^+^‐induced Mitochondrial Dysfunction in SH‐SY5Y Cells

**FIGURE 1:**
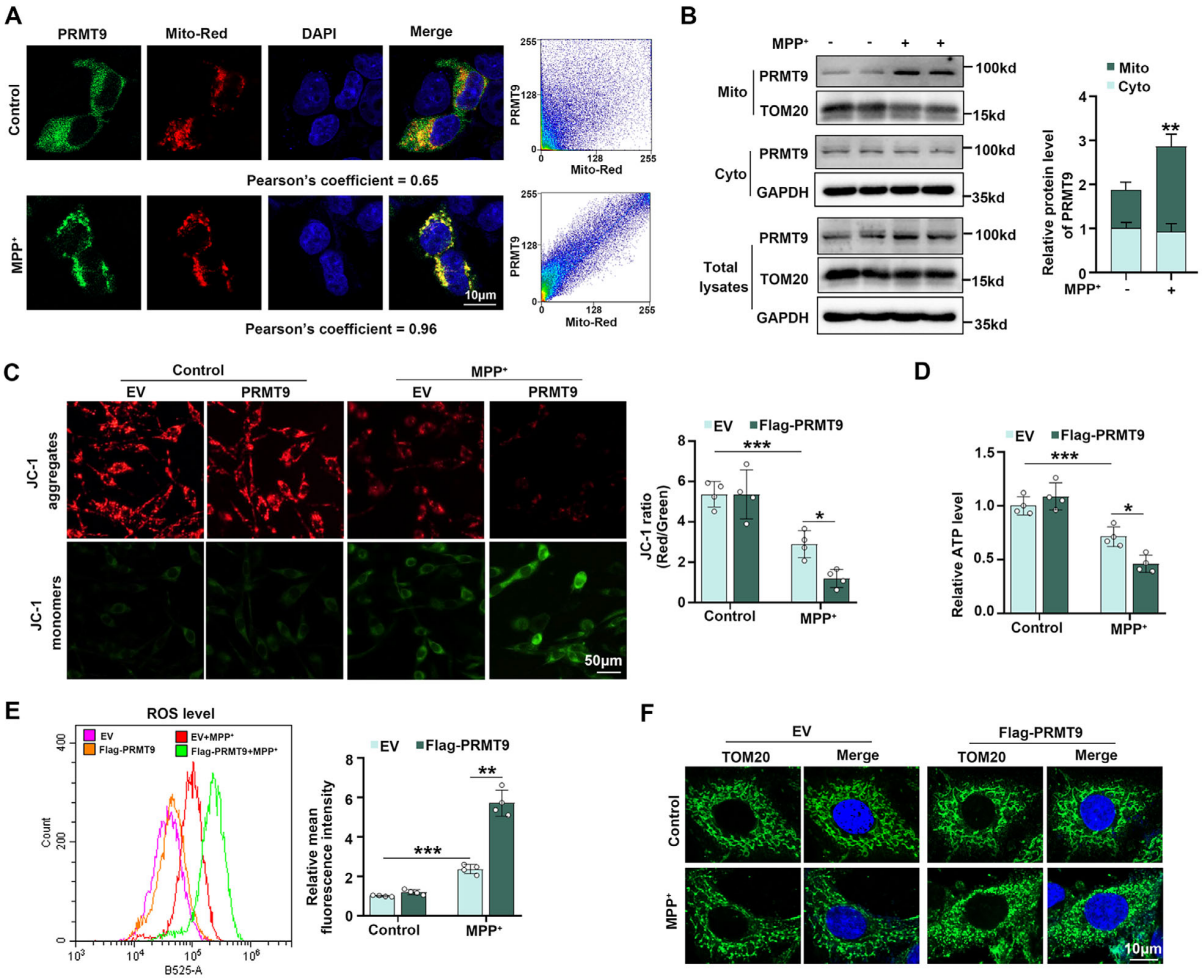
PRMT9 aggravated MPP^+^‐induced mitochondrial dysfunction in SH‐SY5Y cells. HEK 293T cells and SH‐SY5Y cells were treated with MPP^+^ (300 µM) for 24 h respectively. (A) Confocal microscopy of HEK 293T cells transfected with plasmids expressing GFP‐PRMT9 (green) and DsRed2‐Mitored (red). Co‐localization was quantified by using Pearson's correlation coefficient method from Image J software. Scale bars: 10 µm. (B) Western blot analysis of PRMT9 expression in total cell lysates or crude mitochondria lysates from SH‐SY5Y cells with MPP^+^ for 24 h. *n*  =  3 independent experiments. Detection of JC‐1 signals (C) and ATP production (D) in SH‐SY5Y cells under PRMT9 overexpression. (E) Flow Cytometry analysis of ROS production in SH‐SY5Y cells transfected with Flag‐PRMT9 for 24 h of MPP^+^ treatment. (F) Confocal microscopy of mitochondrial fragmentation in SH‐SY5Y cells transfected with Flag‐PRMT9 for 24 h of MPP^+^ treatment. Results were representative of 4 independent experiments. All data are shown as mean ± SD. Multiple comparisons were evaluated by two‐way ANOVA followed by Tukey's test, *p < 0.05, **p < 0.01, ***p < 0.001 compared with indicated group.

Mitochondrial dysfunction is strongly implicated in the etiology of idiopathic and genetic PD [24], and MPTP‐treated mice or MPP^+^‐intoxicated SH‐SY5Y cells are commonly used as PD models [25]. Previous studies revealed that among the PRMTs, only PRMT7 and PRMT9 partially colocalized with mitochondria in macrophages or THP‐1 cells [15]. To identify the potential functions of PRMT7 and PRMT9 in PD, we transfected GFP‐PRMT7 or GFP‐PRMT9 plasmids together with DsRed2‐Mito plasmid into HEK 293T cells followed by MPP^+^ treatment for 24 h, then the mitochondrial localization was measured by confocal microscopic analysis. It was observed that PRMT9 incrementally colocalized with mitochondria upon MPP^+^ stimulation (Figure 1A, Figure S1A), while the colocalization between PRMT7 and mitochondria was unchanged obviously (Figure S1B). Next, crude mitochondria were extracted from SH‐SY5Y cells, and the protein levels of PRMT9 in total cell lysates or mitochondria lysates were obviously elevated (Figure 1B), which was consistent with the results from confocal microscope images.

To clarify whether PRMT9 affected mitochondrial function, we first assessed the collapse of the mitochondrial membrane potential, an essential indicator of mitochondrial dysfunction [26]. JC‐1 analysis revealed that PRMT9 overexpression exacerbated MPP^+^‐induced decrement in mitochondrial membrane potential (Figure 1C). Besides, PRMT9 overexpression further dampened ATP production (Figure 1D) and increased ROS production significantly in SH‐SY5Y cells induced by MPP^+^ (Figure 1E). In addition, MPP^+^ stimulation induced mitochondrial fragmentation, which was exacerbated by PRMT9 overexpression (Figure 1F). Consistently, PRMT9 knockdown stabilized the mitochondrial membrane potential, enhanced ATP production, suppressed the ROS increment and attenuated mitochondrial fragmentation after MPP^+^ treatment (Figure S2). Collectively, PRMT9 translocated to mitochondria and aggravated mitochondrial dysfunction in MPP^+^‐exposed SH‐SY5Y cells.

### 2.2 PRMT9 Exacerbated MPP^+^‐induced Apoptosis in SH‐SY5Y Cells

**FIGURE 2:**
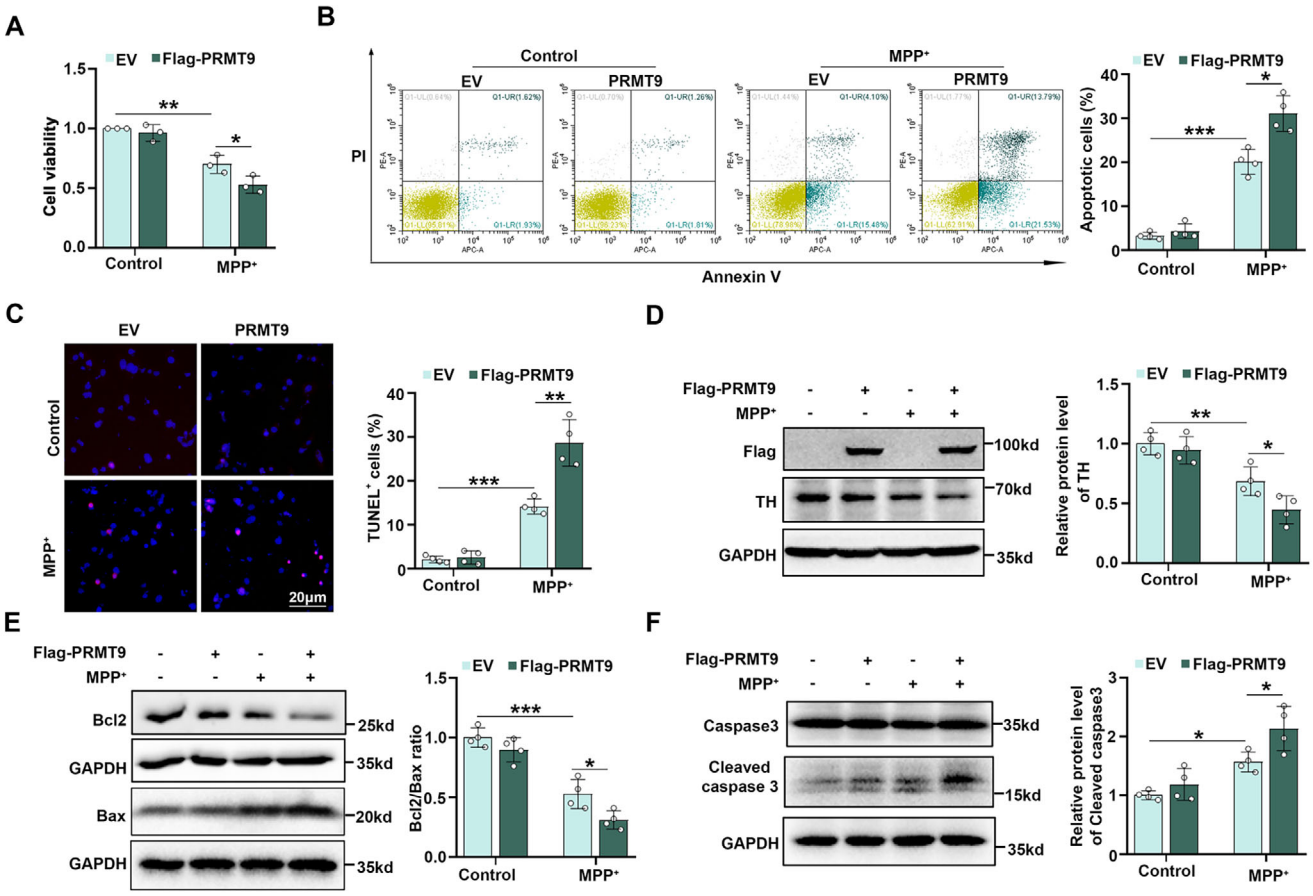
PRMT9 exacerbated MPP^+^‐induced apoptosis in SH‐SY5Y cells. SH‐SY5Y cells transfected with Flag‐PRMT9 were subjected to MPP^+^ (300 µM) for 24 h. (A) Cell viability was determined by CCK‐8 assay. Cell death rate was measured by flow cytometry analysis (B) and TUNEL staining (C). The protein levels of TH (D), Bcl‐2 and BAX (E), Caspase 3 and Cleaved caspase 3 (F) were assessed. Results were representative of 3 or 4 independent experiments. All data are shown as mean ± SD. Multiple comparisons were evaluated by two‐way ANOVA followed by Tukey's test, *p < 0.05, **p < 0.01, ***p < 0.001 compared with indicated group.

Based on our results that PRMT9 regulated mitochondrial homeostasis, we further investigated whether PRMT9 was involved in PD. The results showed PRMT9 overexpression significantly suppressed cell viability of SH‐SY5Y cells treated with MPP^+^ for 24 h (Figure 2A) while PRMT9 silence improved the cell viability (Figure S3A). Apoptosis has been extensively demonstrated in PD patients and experimental models [27]. Flow cytometry analysis and TUNEL staining results revealed that PRMT9 overexpression markedly increased cell death induced by MPP^+^ (Figure 2B,C) while PRMT9 silence restrained the ratio of apoptotic or TUNEL^+^ cells stimulated with MPP^+^ (Figure S3B,C). In addition, Tyrosine hydroxylase (TH), a marker of DA neuronal viability, catalyzes the first and rate‐limiting step in the biosynthesis of dopamine and other catecholamines [28]. We found PRMT9 overexpression further aggravated MPP^+^‐induced TH impairment (Figure 2D). In contrast, PRMT9 knockdown greatly suppressed the reduction of TH in SH‐SY5Y cells induced by MPP^+^ (Figure S3D). Moreover, PRMT9 overexpression notably reduced Bcl2/Bax ratio and enhanced Caspase 3 cleavage upon MPP^+^ stimulation in SH‐SY5Y cells (Figure 2E,F), which indicated PRMT9 overexpression cells were more susceptible to MPP^+^‐induced apoptosis than control cells. Consistently, PRMT9 silence significantly ameliorated MPP^+^‐induced cell apoptosis in SH‐SY5Y cells evidenced with the increment of Bcl2/Bax ratio (Figure S3E) and the reduction of cleaved Caspase 3 (Figure S3F).

### 2.3 PRMT9 Conditional Deficiency Ameliorated Dopaminergic Degeneration Induced by MPTP

**FIGURE 3:**
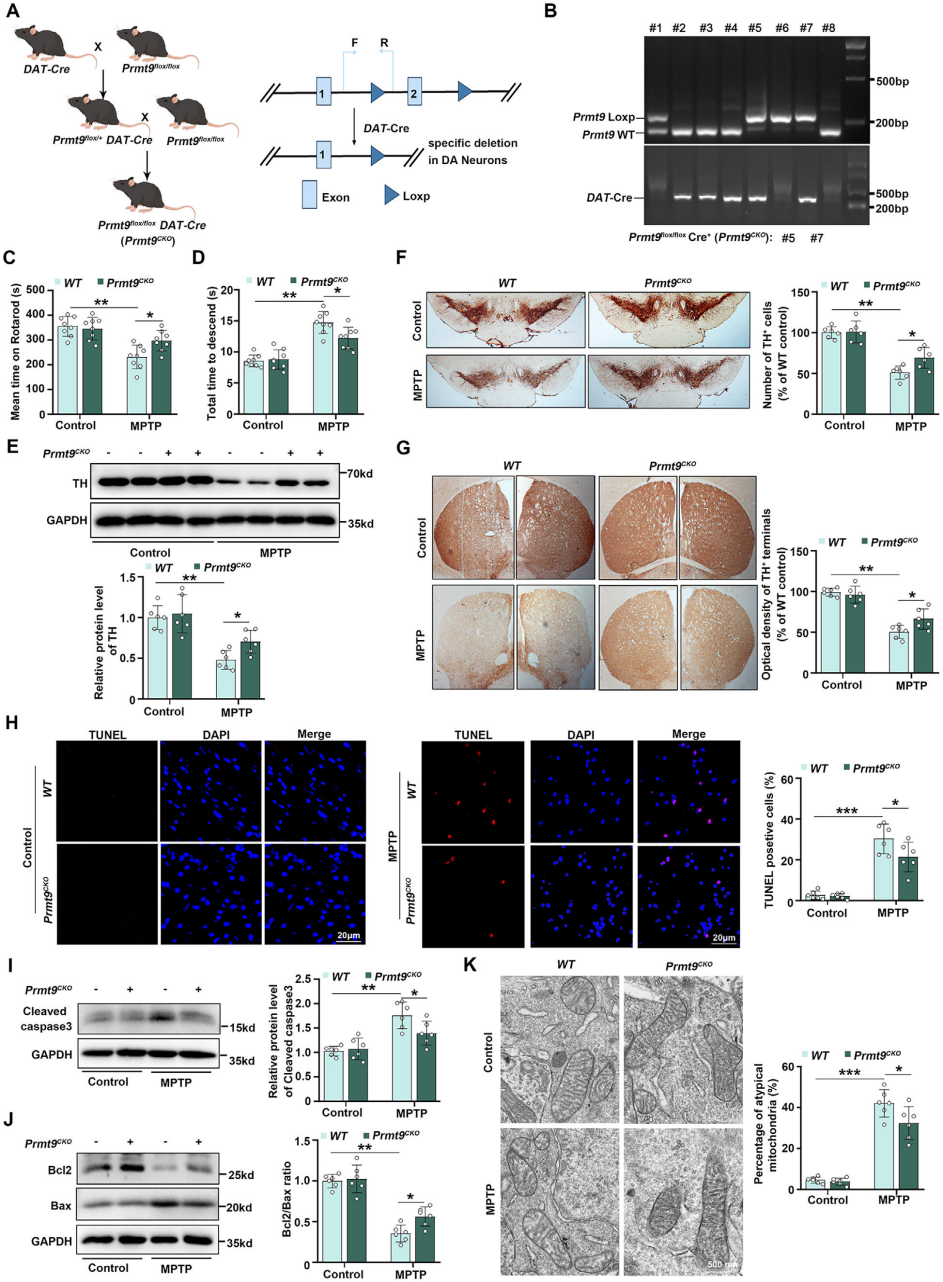
PRMT9 conditional deficiency ameliorated dopaminergic degeneration induced by MPTP. (A) A diagram for the generation of PRMT9 knockout in dopaminergic (DA) neurons (*Prmt9^CKO^* mice). (B) PCR was performed on genomic DNA extracted from mice tail to confirm PRMT9 knockout in DA neurons. WT and *Prmt9^CKO^* mice were injected with 20 mg kg^−1^ MPTP 4 times a day, with a 2 h interval, for duration of 7 days. (C) Rotarod test and (D) pole test were used to evaluate the motor coordination on mice subjected to MPTP treatment (*n* = 8 mice per group). Western blot analysis of lysates from SN of WT or *Prmt9^CKO^* mice treated with MPTP. The protein levels of TH (E), Cleaved caspase 3 (I), Bcl‐2 and BAX (J) were assessed (*n* = 6 mice per group). (F, G) Micrographs of DA neurons in SN or TH fiber density in STR of WT or *Prmt9^CKO^* mice treated with MPTP by TH staining (*n* = 6 mice per group). (H) Representative images and quantification of TUNEL staining in SN of WT or *Prmt9^CKO^* mice treated with MPTP. Scale bars, 20 µm. (*n* = 6 mice per group). (K) TEM micrograph of mitochondria in SN of WT or *Prmt9^CKO^* mice treated with MPTP (*n* = 6 mice per group). All data are shown as mean ± SD. Multiple comparisons were evaluated by two‐way ANOVA followed by Tukey's test, *p < 0.05, **p < 0.01, ***p < 0.001 compared with indicated group.

To further elucidate the potential role of PRMT9 in the development of PD, we bred *Prmt9^flox/flox^/Cre^+^* (*Prmt9^CKO^*) mice to knockout PRMT9 specifically in DA neurons (Figure 3A,B). Littermate *Prmt9^flox/flox^/Cre^−^* mice were used as wild‐type (WT) controls. The efficiency of PRMT9 knockout was assessed by Western blot analysis in SN tissues (Figure S4A). Motor behavior of WT and *Prmt9^CKO^* mice was evaluated by the rotarod and pole tests after MPTP administration. As shown in Figure 3C, *Prmt9^CKO^* mice treated with MPTP exhibited a longer latency to fall from the rod than WT mice. In the pole test, the total time that *Prmt9^CKO^* mice spent on the pole was obviously shortened after MPTP administration compared with WT mice (Figure 3D). These data suggested that the deletion of PRMT9 in DA neurons ameliorated MPTP‐induced motor deficits. In addition, Western blot results showed that the reduction of TH protein level in SN and STR induced by MPTP was partially reverted by PRMT9 conditional knockout (Figure 3E, Figure S4B). Consistently, immunohistochemistry staining confirmed that PRMT9 deficiency strongly improved the loss of TH‐positive neurons in the SN and TH‐positive fibers in the STR (Figure 3F,G). Moreover, *Prmt9^CKO^* mice displayed an obvious increase of Bcl2/Bax ratio and an evident reduction of cleaved Caspase 3 protein level in SN and STR after MPTP treatment compared with WT mice (Figure 3I,J, Figure S4C). It was also observed that PRMT9 deficiency reduced the ratio of TUNEL‐positive cells in SN tissues of mice upon MPTP stimulation (Figure 3H). Besides, the transmission electron microscope (TEM) results showed that PRMT9 deficiency ameliorated morphological defects in mitochondrial ultrastructure, including mitochondrial vacuolization, membrane ruffling and cristae blurring compared to WT mice after MPTP treatment (Figure 3K). Taken together, these results demonstrated that PRMT9 deficiency ameliorated DA degeneration induced by MPTP.

### 2.4 Overexpression of PRMT9 Aggravated MPTP‐induced Dopaminergic Degeneration

**FIGURE 4:**
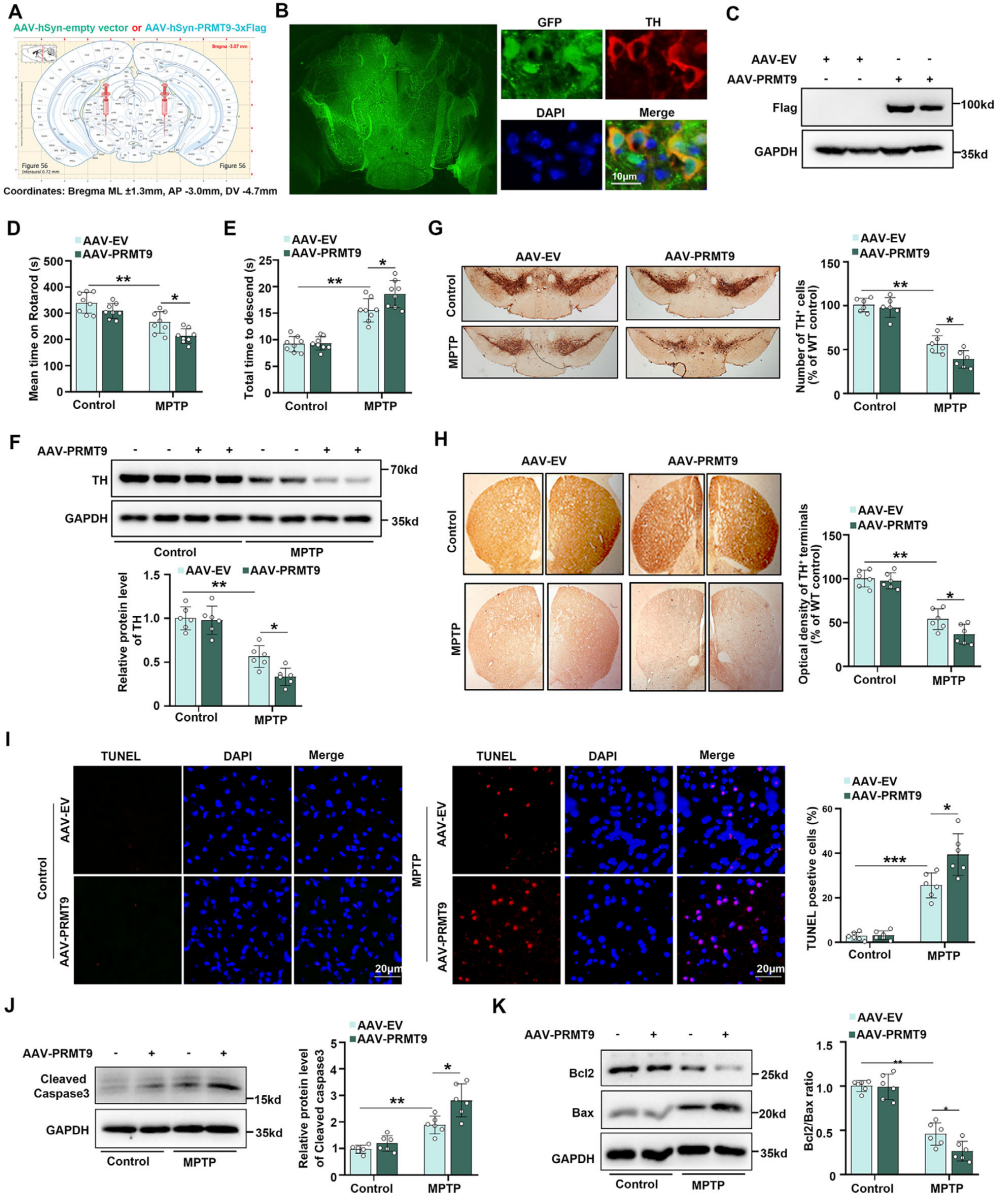
Overexpression of PRMT9 aggravated MPTP‐induced dopaminergic degeneration. Adeno‐associated virus (AAV) containing PRMT9 (AAV‐PRMT9) was injected into the SN of WT mice for 3 weeks. Then the mice were subjected to MPTP treatment. (A) The injection coordinates. The AAV‐PRMT9 efficiently infected mouse brain (B) and the protein expression of PRMT9 (C) was elevated in the AAV‐PRMT9‐injected mice. WT and AAV‐PRMT9‐injected mice were injected with 20 mg kg^−1^ MPTP 4 times a day, with a 2 h interval, for duration of 7 days. (D) Rotarod test and (E) pole test were used to evaluate the motor coordination on mice subjected to MPTP treatment (*n* = 8 mice per group). Western blot analysis of lysates from SN of WT or PRMT9 overexpressed mice treated with MPTP. The protein levels of TH (F), Cleaved caspase 3 (J), Bcl‐2 and BAX (K) were assessed (*n* = 6 mice per group). (G, H) Micrographs of DA neurons in SN or TH fiber density in STR of WT or PRMT9 overexpressed mice treated with MPTP by TH staining (*n* = 6 mice per group). (I) Representative images and quantification of TUNEL staining in SN of WT or PRMT9 overexpressed mice treated with MPTP. Scale bars, 20 µm (*n* = 6 mice per group). All data are shown as mean ± SD. Multiple comparisons were evaluated by two‐way ANOVA followed by Tukey's test, *p < 0.05, **p < 0.01, ***p < 0.001 compared with indicated group.

To prove the function of PRMT9 in the process of PD, we overexpressed PRMT9 by injecting AAV‐PRMT9 into the SN of WT mice and the control group was injected with AAV‐Vector to avoid any non‐specific effects of viral injection. The neuron‐specific promoter, human Synapsin I (hSyn), was utilized in AAV‐PRMT9 construct for high expression of PRMT9 (Figure 4A) [29, 30]. The efficiency of PRMT9 overexpression was evaluated by GFP fluorescence in brain sections (Figure 4B) and Western blot analysis in SN tissues (Figure 4C). Moreover, overexpressed PRMT9 indeed existed in TH‐positive DA neurons (Figure 4B). As shown in Figure 4D,E, PRMT9 overexpression significantly aggravated MPTP‐induced behavioral deficits. Additionally, the decrease of TH expression in SN and STR induced by MPTP was further enhanced in the PRMT9 overexpression mice (Figure 4F, Figure S5), which was consistent with the immunohistochemistry results shown in Figure 4G,H. Meanwhile, the Bcl2/Bax ratio was evidently decreased and the cleaved Caspase 3 and the percentage of TUNEL‐positive cells were increased in PRMT9 overexpression mice compared with WT mice after MPTP execution (Figure 4I–K). Thus, these data strongly suggested PRMT9 overexpression aggravated MPTP‐induced dopaminergic degeneration.

To further provide human genetic evidence supporting a causal link between PRMT9 and PD pathogenesis, we performed two‐sample Mendelian Randomization (MR) analysis. We obtained cis‐expression quantitative trait loci (cis‐eQTL) summary statistics for PRMT9 in human SN tissues from the Genotype‐Tissue Expression (GTEx) database as genetic instrumental variables and extracted the association estimates between these instruments and PD risk from large‐scale PD genome‐wide association study (GWAS) summary data. Our MR results demonstrated that genetically elevated PRMT9 expression in the SNpc was significantly associated with increased risk of PD (*p* < 0.05), supporting the clinical relevance of PRMT9 in PD pathogenesis (Figure S6).

### 2.5 PRMT9 Was Upregulated in PD Mice and Cell Models Induced by MPTP or MPP^+^ Respectively, Which Negatively Correlated With DUSP26

**FIGURE 5:**
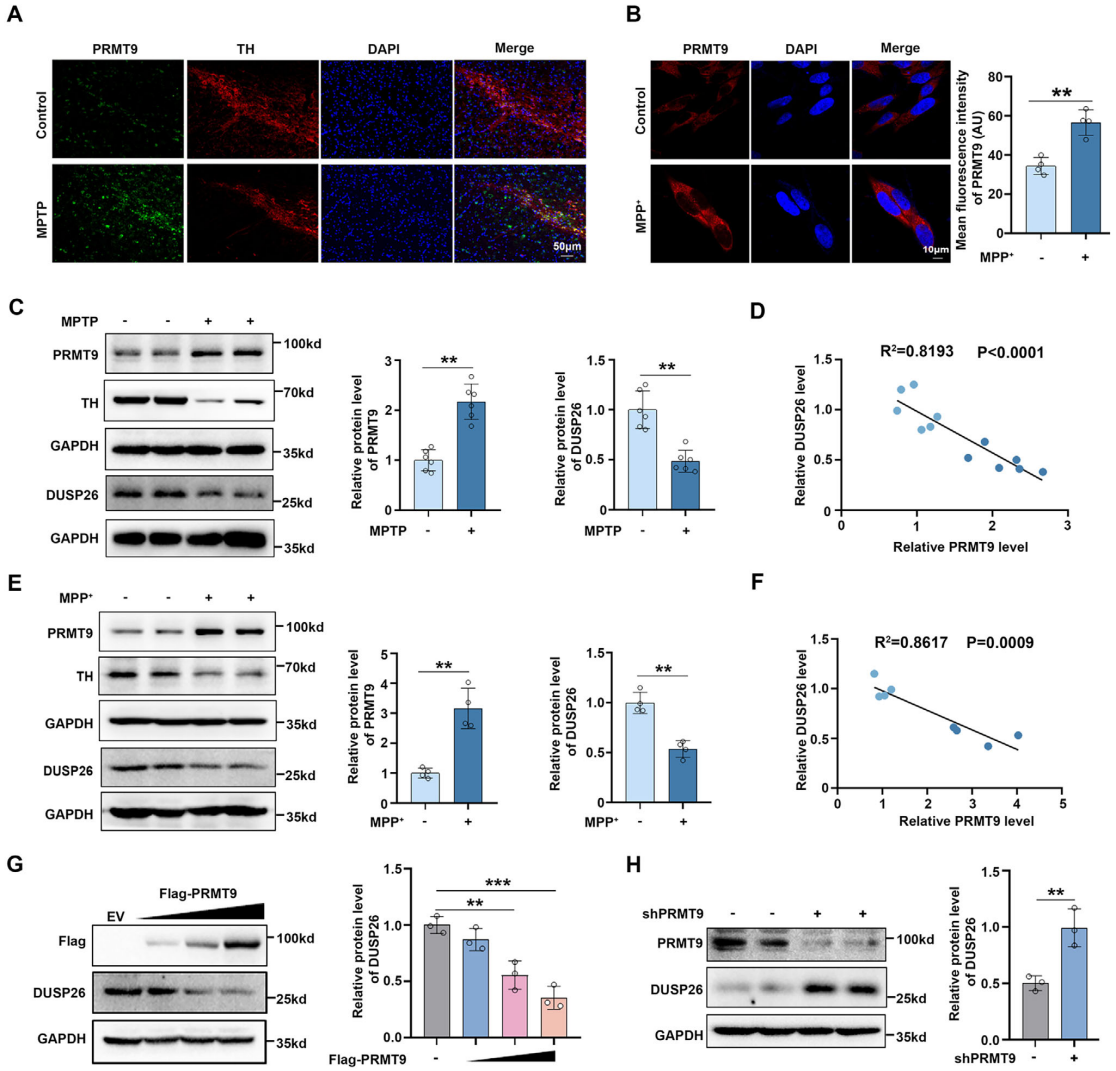
The protein level of PRMT9 was upregulated in PD mice and cell model induced by MPTP or MPP^+^ respectively, which negatively correlated with DUSP26. WT mice were injected with 20 mg kg^−1^ MPTP 4 times a day, with a 2 h interval, for duration of 7 days. SH‐SY5Y cells were treated with MPP^+^ (300 µM) for 24 h. (A) Double immunofluorescence of PRMT9 (green) and TH (DA neuron marker, red) were performed in SN of mouse brain after MPTP treatment. Scale bars, 50 µm. (B) Immunofluorescence of PRMT9 (green) was performed in SH‐SY5Y cells. Scale bars, 10 µm. (C) The protein levels of TH, PRMT9 and DUSP26 were assessed in SN of WT mice subjected to MPTP, *n* = 6 mice per group. (D) Pearson correlation analyses showing the correlations between PRMT9 and DUSP26 expression in SN of WT mice subjected to MPTP. (E) The protein levels of TH, PRMT9 and DUSP26 were assessed in SH‐SY5Y cells subjected to MPP^+^, *n* = 4 independent experiments. (F) Pearson correlation analyses showing the correlations between PRMT9 and DUSP26 expression in SH‐SY5Y cells subjected to MPP^+^. (G, H) Western blot analysis on the expression of DUSP26 in lysates of SH‐SY5Y cells after gradient transfection of GFP‐PRMT9 plasmid or shPRMT9. *n* = 3 independent experiments. All data was expressed as the mean ± SD. Two‐tailed Student's *t* test or one‐way ANOVA was used for statistical analyses. *p < 0.05, **p < 0.01, ***p < 0.001 compared with indicated group.

To investigate the underlying mechanisms by which PRMT9 contributed to PD, we first evaluated the protein levels of PRMT9 in PD models. We found PRMT9 was upregulated and mainly detected in TH‐positive neurons in the SN of MPTP‐induced mouse models by immunofluorescence (Figure 5A). PRMT9 protein level was also increased in SH‐SY5Y cells and cultured midbrain primary neurons after MPP^+^ (Figure 5B,E, Figure S7A). Similar results were observed in SN and STR of MPTP‐induced mice models (Figure 5C, Figure S7B). Additionally, the upregulation of PRMT9 was observed in SH‐SY5Y cells (Figure S8A) and SN of mice (Figure S8B) treated by α‐Syn PFFs. Moreover, the transcription levels of PRMT9 were significantly upregulated in the PD models induced by MPP^+^ (Figure S9A,B). Consistently, analysis of the public GEO dataset GSE168496 revealed that PRMT9 mRNA levels were significantly increased in the SNpc of PD patients compared with healthy controls (Figure S9C). Notably, in other neurodegenerative disorders associated with mitochondrial dysfunction, including Alzheimer's disease (AD, GSE285831) and Huntington's disease (HD, GSE220847), PRMT9 expression showed no significant changes in brain tissues (Figure S9D,E). Moreover, we assessed the protein level of DUSP26, which was a key regulator of mitochondrial homeostasis and convincingly participated in the pathological process of PD [21, 23]. DUSP26 was recently identified as a potential interacting protein of PRMT9 [31]. Our results showed DUSP26 was downregulated in PD mice and cell models (Figure 5C,E, Figure S7B) which was consistent with the transcription data from the GEO database that DUSP26 expression in PD patients was decreased compared with control donors (Figure S10). Importantly, the linear regression analysis displayed a negative correlation between PRMT9 and DUSP26 expression at both the tissue and cellular levels (Figure 5D,F, Figure S7C). To clarify the temporal relationship between PRMT9 upregulation and DUSP26 downregulation, we performed a time‑course analysis in SH‑SY5Y cells exposed to MPP^+^. PRMT9 protein level was significantly increased as early as 6 h after MPP^+^ treatment, whereas DUSP26 protein showed an obvious reduction until 12 h (Figure S11). This temporal order indicated that PRMT9 upregulation occurred prior to DUSP26 loss, supporting that PRMT9 might act as an upstream driver to reduce DUSP26 in PD models.

Based on the aforementioned results that PRMT9 was negatively correlated to DUSP26 expression and presumed to be an interactive partner of DUSP26, we further determined whether PRMT9 regulated DUSP26. The result showed that overexpression of Flag‐tagged PRMT9 induced a dramatic decrease of DUSP26 in a dose‐dependent manner both in SH‐SY5Y (Figure 5G) and HEK 293T cells (Figure S12A). Consistently, DUSP26 protein level was elevated when PRMT9 was knocked down using shRNA (Figure 5H) and siRNA (Figure S12B) respectively. These data indicated that PRMT9 may be involved in PD by targeting DUSP26.

### 2.6 PRMT9 Involved in PD by Promoting the Proteasomal Degradation of DUSP26

**FIGURE 6:**
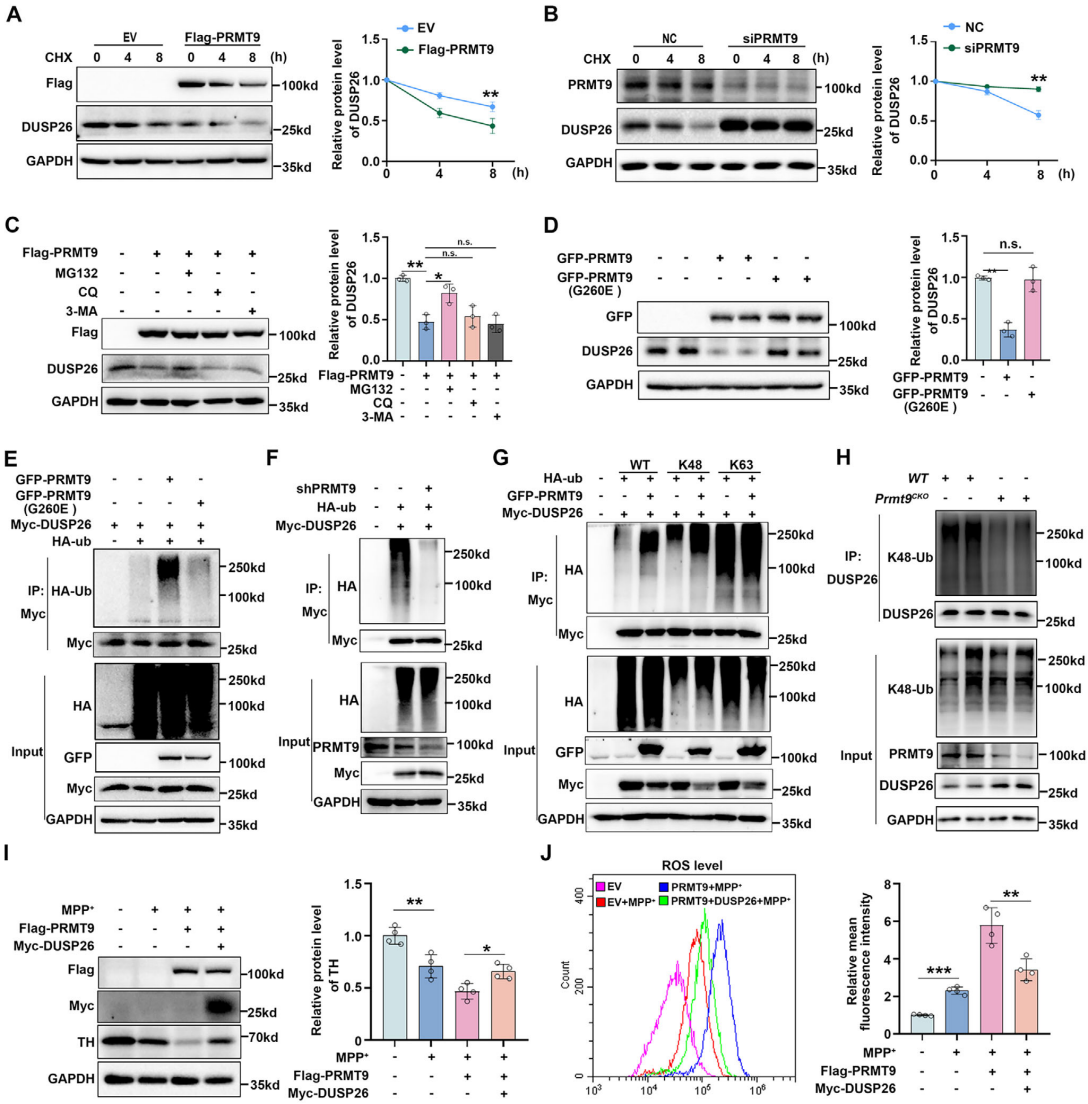
PRMT9 involved in PD by promoting the proteasomal degradation of DUSP26. (A, B) HEK 293T cells were transfected with Flag‐PRMT9 expression plasmid or siPRMT9. The transfected cells were cultured for 36 h before being incubated with cycloheximide (CHX) for the indicated time. The levels of DUSP26 in cells lysates at different time points were detected by Western blot analysis. *n* = 3 independent experiments. (C) Western blot analysis of extracts from HEK 293T cells transfected with Flag‐PRMT9 expression plasmid then treated with MG132 (10 µM), Chloroquine (10 µM) or 3‐MA (10 mM) for 8 h. (D) Western blot analysis of DUSP26 in SH‐SY5Y cells transfected with GFP‐PRMT9 plasmid (WT or G260E). (E, F) IP analysis of DUSP26 ubiquitination in HEK 293T cells transfected with plasmids expressing Myc‐DUSP26, HA‐Ub, as well as GFP‐PRMT9 (WT), GFP‐PRMT9 (G260E) or shPRMT9. (G) IP analysis of DUSP26 ubiquitination in HEK 293T cells transfected with plasmids expressing Myc‐DUSP26, GFP‐PRMT9, HA‐Ub and its mutants that can only append either K48 or K63 ubiquitin chains. (H) IP analysis of DUSP26 ubiquitination in SN lysates from WT and *Prmt9^CKO^mice*. IP with anti‐DUSP26 antibody, probed with anti‐K48‐Ub antibody. In rescue experiments, Flag‐PRMT9 and Myc‐DUSP26 plasmids were co‐transfected into SH‐SY5Y cells upon 300 µM MPP^+^ stimulation. The protein levels of TH (I) were assessed. ROS production (J). Results were representative of 3 or 4 independent experiments. All data was expressed as the mean ± SD. One‐way ANOVA was used for statistical analyses. *p < 0.05, **p < 0.01, ***p < 0.001 compared with indicated group.

To elucidate how PRMT9 regulated DUSP26 protein level, we performed qPCR to assess potential changes in DUSP26 mRNA levels following PRMT9 modulation. There was no effect of PRMT9 overexpression or knockdown on DUSP26 transcription (Figure S13A–D). Then we assumed that PRMT9 may regulate the protein level of DUSP26 by promoting its degradation [32, 33]. We inhibited de novo protein synthesis by cycloheximide (CHX) to evaluate the influence of PRMT9 on DUSP26 degradation. The results showed PRMT9 significantly promoted DUSP26 protein degradation (Figure 6A). Conversely, DUSP26 degradation was attenuated in PRMT9 knockdown SH‐SY5Y cells (Figure 6B). We then explored the degradation pathway of DUSP26 mediated by PRMT9. The degradation of DUSP26 induced by PRMT9 was reversed by proteasome inhibitor MG132, but not by lysosome inhibitor chloroquine (CQ) or autophagy inhibitor 3‐MA, indicating that PRMT9 promoted DUSP26 degradation by the proteasomal pathway (Figure 6C). Mutation at Gly260 was reported to render PRMT9 into a catalytically inactive form [34]. Then, we assessed whether PRMT9 regulated the degradation of DUSP26 depending on its enzymatic activity. Notably, we observed that WT PRMT9, rather than mutant PRMT9 (G260E), impaired DUSP26 stability, indicating that the arginine methyltransferase activity of PRMT9 was required for the degradation of DUSP26 (Figure 6D). A great deal of studies indicated that ubiquitination was identified as a key regulatory feature in protein degradation. K48‐linked polyubiquitin chains are the canonical signal for proteasomal degradation, while K63‐linked chains are mainly involved in signal transduction and protein trafficking [35, 36]. Next, Myc‐tagged DUSP26, HA‐tagged ubiquitin and GFP‐tagged PRMT9 or PRMT9 mutant G260E were co‐transfected into HEK 293T cells. The ubiquitination of DUSP26 was significantly enhanced by WT PRMT9 rather than the PRMT9 (G260E) mutant (Figure 6E). Consistently, PRMT9 knockdown greatly reduced the ubiquitination level of DUSP26 in HEK 293T cells (Figure 6F). To clarify which type of ubiquitin chain attached to DUSP26, GFP‐PRMT9, Myc‐DUSP26 and K48 or K63 ubiquitin plasmids (containing only K48 or K63‐linked polyubiquitin chains respectively) were co‐transfected into HEK 293T cells. As shown in Figure 6G, PRMT9 promoted the K48‐linked but not K63‐linked polyubiquitin chains of DUSP26. Consistently, the endogenous K48‐linked polyubiquitination levels of DUSP26 in the SN of *Prmt9^CKO^* mice showed a significant reduction compared to *WT* controls (Figure 6H).

To verify whether PRMT9 was involved in the process of PD by regulating DUSP26, we monitored DUSP26 and its downstream signals in PD models. As well established in PD, p38 MAPK serves as a pro‑apoptotic kinase that drives DA neuronal death [37, 38, 39, 40]. Functionally, DUSP26 serves as a specific phosphatase for p38 and negatively modulates p38 activation in PD [23]. In our results, PRMT9 overexpression downregulated DUSP26 protein level and activated p‐p38 in PD cell models (Figure S14A), and the effect was confirmed by PRMT9 silence results (Figure S14B). Furthermore, consistent results were obtained in SN of PD mouse models with PRMT9 knockout or overexpression (Figure S15A,B). Next, Myc‐tagged DUSP26 and Flag‐tagged PRMT9 plasmids were co‐transfected into SH‐SY5Y cells. After 24 h of MPP^+^ treatment, the PRMT9 overexpression cells showed a further loss of TH protein, whereas enhanced DUSP26 expression alleviated the neurotoxicity induced by PRMT9 overexpression in MPP^+^‐treated SH‐SY5Y cells (Figure 6I). Moreover, the further increase of ROS induced by PRMT9 overexpression under MPP^+^ stimulation was markedly abolished by elevated expression of DUSP26 (Figure 6J). Additionally, DUSP26 overexpression markedly rescued the cell viability (Figure S16A) and cell apoptosis induced by PRMT9 overexpression in MPP^+^‐treated SH‐SY5Y cells assessed by TUNEL staining (Figure S16B) and flow cytometry analysis (Figure S16C). Collectively, these data indicated that PRMT9 exacerbated MPP^+^‐induced damage in SH‐SY5Y cells through the DUSP26‑p38 signaling axis.

### 2.7 PRMT9 Interacted With DUSP26 and Catalyzed Its Arginine Methylation at R29

**FIGURE 7:**
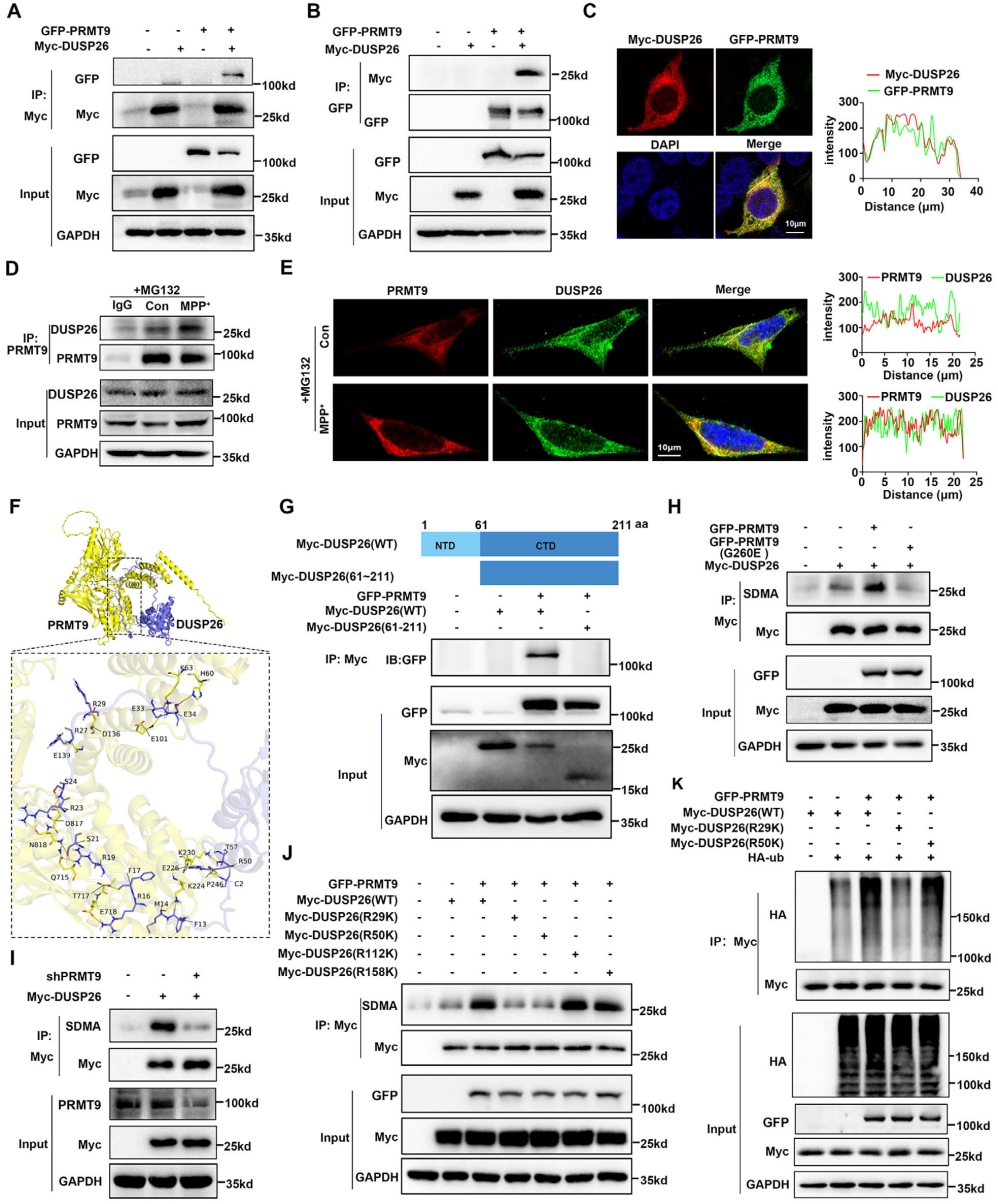
PRMT9 interacted with DUSP26 and promoted its arginine methylation at residue R29. (A, B) Co‐IP analysis of the exogenous interaction of PRMT9 with DUSP26 in HEK 293T cells transfected with plasmids expressing GFP‐PRMT9 and Myc‐DUSP26. (C) Representative images of laser scanning confocal microscopy for GFP‐PRMT9 (green) and Myc‐DUSP26 (red) in HEK 293T cells. Scale bars, 5 µm. (D) Co‐IP analysis of the endogenous interaction of PRMT9 with DUSP26 in SH‐SY5Y cells subjected to 24 h MPP^+^ (300 µM) treatment. (E) Confocal analysis of the co‐localization of endogenous PRMT9 (red) and DUSP26 (green) in SH‐SY5Y cells stimulated by MPP^+^ (300 µM) treatment. Scale bar, 10 µm. (F) Molecular docking using HADDOCK software predicted the interaction between PRMT9 and DUSP26. (G) Myc‐tagged DUSP26 (WT) or its truncated mutants and GFP‐PRMT9 were individually transfected into HEK 293T cells. The cell lysates were immunoprecipitated with an anti‐Myc antibody and then immunobloted with the indicated antibody. (H, I) IP analysis of the exogenous DUSP26 SDMA in HEK 293T cells transfected with Myc‐DUSP26 and GFP‐PRMT9 plasmids or shPRMT9. (J) IP analysis of the SDMA level of DUSP26 in HEK 293T cells transfected with GFP‐PRMT9 and Myc‐DUSP26 plasmids (WT or arginine mutants). (K) IP analysis of DUSP26 ubiquitination in HEK 293T cells transfected with plasmids expressing GFP‐PRMT9, Myc‐DUSP26 (WT or indicated mutants) and HA‐UB.

According to a previous study that PRMT9 was presumed to be an interactive partner of DUSP26 [31], we confirmed whether DUSP26 was a substrate of PRMT9 in PD development. The Co‐IP results clearly showed that PRMT9 interacted with DUSP26 (Figure 7A,B). Meanwhile, immunofluorescence results suggested obvious co‐localization between PRMT9 and DUSP26 (Figure 7C). Consistently, the interaction between endogenous PRMT9 and DUSP26 was observed in both primary midbrain neurons and SH‐SY5Y cells, and this interaction was further enhanced after MPP^+^ stimulation (Figure 7D,E and Figure S17). Furthermore, we purified recombinant proteins and the pull‐down experiment in vitro confirmed the direct interaction between PRMT9 and DUSP26 (Figure S18A). DUSP26 composed of an N‐terminal domain (amino acids 1–60) and a C‐terminal catalytically active domain (amino acids 61–211) [19]. To determine which domain(s) of DUSP26 was necessary for interaction with PRMT9, molecular docking using HADDOCK software successfully generated a structural model of the PRMT9‐DUSP26 complex. This model predicted a favorable binding free energy of ‐16.6 kcal mol^−1^, indicating a stable interaction. Analysis of the binding interface reveals that the primary interaction site is localized within the N‐terminal domain of DUSP26, which engages with PRMT9 through a network of interfacial contacts (Figure 7F). To validate the aforementioned findings, Myc‐tagged DUSP26 truncated mutants were constructed. Co‐IP results showed that DUSP26 truncated mutant lacking the N‐terminal domain lost the ability to interact with PRMT9, indicating the necessity of the N‐terminal domain for the interaction (Figure 7G). Overall, these data indicated that PRMT9 directly targeted DUSP26.

As a type II arginine methyltransferase, PRMT9 catalyzed the SDMA modification in target proteins [15]. Hence, we investigated whether PRMT9 could catalyze DUSP26 arginine methylation. Myc‐DUSP26 plasmid was transfected into HEK 293T cells and the arginine methylation of DUSP26 was readily detected (Figure S18B). Given that PRMT5 and PRMT9 both belonged to type II arginine methyltransferase, we then determined whether the SDMA modification of DUSP26 was partially derived from PRMT5. Notably, Co‐IP results revealed that PRMT5 did not interact with DUSP26, indicating that the formation of SDMA in DUSP26 was entirely derived from PRMT9 (Figure S18C). Next, we further proved that WT PRMT9 rather than its enzymatic mutant enhanced the SDMA formation of DUSP26 (Figure 7H). Consistently, PRMT9 knockdown dramatically reduced the SDMA level of DUSP26 (Figure 7I). To clarify the potential arginine residues in DUSP26 catalyzed by PRMT9, we enriched Myc‐tagged DUSP26 protein in HEK 293T cells and applied liquid chromatography‐mass spectrometry (LC‐MS) analysis to identify the methylated residues. The results showed four arginine (R) residues of DUSP26 were identified as potential methylation sites (Figure S19A). To verify the major methylation sites in DUSP26, we substituted R29, R50, R112, and R158 residues in Myc‐DUSP26 with lysine (K) individually and examined the methylation status of DUSP26. We found only DUSP26 (R29K) and DUSP26 (R50K) mutations led to significant reduction in DUSP26 methylation in the presence of PRMT9 (Figure 7J). The methylation sites prediction analysis (http://msp.biocuckoo.org/) also indicated R29 was the most likely candidate for a methylation site (Figure S19B). Besides, the R29 methylation site on DUSP26 is also highly conserved in mammals (Figure S19C). To verify that PRMT9 promoted the ubiquitination of DUSP26 depending on its arginine methyltransferase activity, DUSP26 (R29K) and DUSP26 (R50K) mutation plasmids were transfected into HEK 293T cells together with GFP‐PRMT9 and HA‐Ub, and the IP results suggested that R29K rather than R50K mutation abolished the DUSP26 ubiquitination induced by PRMT9 (Figure 7K). Consistent with these findings, molecular docking analysis of the binding interface revealed that the R29 residue of DUSP26 was situated within the core interaction interface with PRMT9 and was physically accessible to the catalytic domain of PRMT9 (Figure 7F). This structural observation provides a solid mechanistic basis for PRMT9‐mediated R29 methylation of DUSP26. Furthermore, we found that the PRMT9 (G260E) mutant still interacted with DUSP26, indicating that the regulation of DUSP26 stability is dependent on PRMT9‐mediated methylation rather than its physical binding alone (Figure S20A). Besides, the CHX chase assay showed that the DUSP26‐R29K mutant was significantly more stable than WT‐DUSP26 (Figure S20B). Taken together, our results demonstrated that PRMT9‐mediated meR29‐DUSP26 impaired the stability of DUSP26 by promoting its ubiquitination.

### 2.8 PRMT9 Promoted the Degradation of DUSP26 Mediated by E3 Ligase Trim32

**FIGURE 8:**
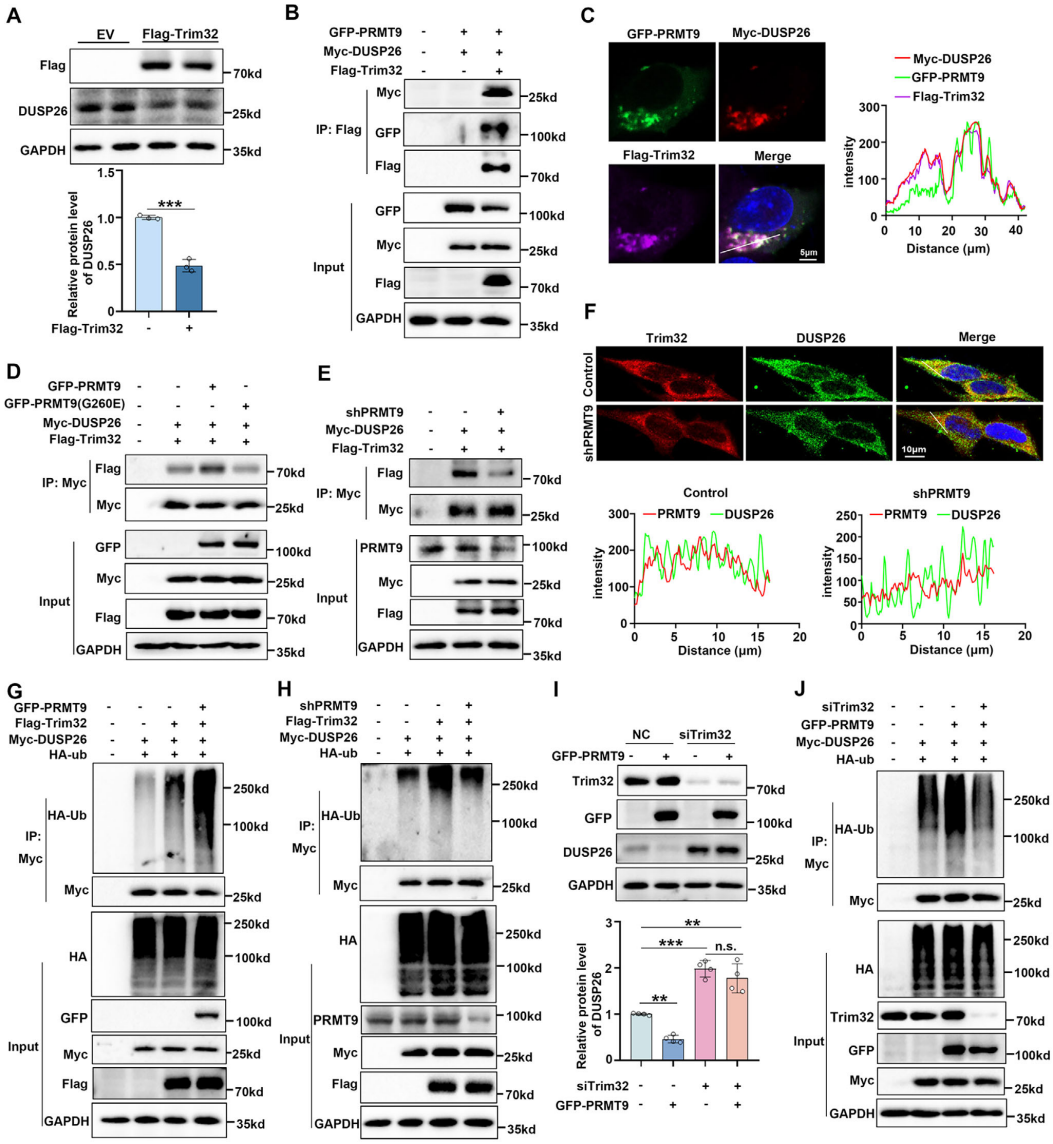
PRMT9 promoted the degradation of DUSP26 mediated by E3 ligase Trim32. (A) Western blot analysis on the expression of DUSP26 in lysates of HEK 293T cells after transfection of Flag‐Trim32 plasmid. *n* = 3 independent experiments. (B) Co‐IP analysis of the exogenous interaction of Trim32, DUSP26 and PRMT9 in HEK 293T cells transfected with plasmids expressing Flag‐Trim32, GFP‐PRMT9 and Myc‐DUSP26. (C) Representative images of laser scanning confocal microscopy for GFP‐PRMT9 (green), Myc‐DUSP26 (red) and Flag‐Trim32 (purple) in HEK 293T cells. Scale bars, 5 µm. (D‐E) Co‐IP analysis of the exogenous interaction strength between Trim32 and DUSP26 in HEK 293T cells transfected with Flag‐Trim32 and Myc‐DUSP26 plasmids under PRMT9 overexpression or knockdown condition. (F) Confocal analysis of the endogenous interaction strength between Trim32 (red) and DUSP26 (green) in SH‐SY5Y cells stimulated under by PRMT9 knockdown condition. Scale bar, 10 µm. (G‐H) IP analysis of DUSP26 ubiquitination in HEK 293T cells transfected with Flag‐Trim32 and Myc‐DUSP26 plasmids under PRMT9 overexpression or knockdown condition. (I) Western blot analyses of DUSP26 in HEK 293T cells transfected with GFP‐PRMT9 plasmids and siTrim32. (J) IP analysis of DUSP26 ubiquitination in HEK 293T cells transfected with GFP‐PRMT9 plasmids and siTrim32.

To gain further insights into the specific mechanism by which PRMT9 promoted DUSP26 degradation, we screened out the potential E3 ubiquitin ligases Trim32 and LTN1 (Figure S21A), which may interact with DUSP26 based on its LC‐MS data [31] and exhibited significant associations with PD [41, 42]. We subsequently evaluated the impact of LTN1 and Trim32 overexpression on DUSP26 protein abundance. Our results revealed that LTN1 overexpression had no significant effect on DUSP26 levels (Figure S21B). In contrast, Trim32 overexpression markedly reduced DUSP26 protein abundance (Figure 8A), mirroring the effect observed with PRMT9. Therefore, we next investigated whether the degradation of DUSP26 relied on Trim32. Molecular docking demonstrated a stable interaction between Trim32 and DUSP26, with a binding free energy of ‐11.2 kcal mol^−1^ (Figure S22A). Co‐IP results revealed that Trim32 interacted with both PRMT9 and DUSP26 exogenously (Figure 8B) and confocal microscopy further validated the interaction among them (Figure 8C). Interestingly, we found that WT PRMT9 significantly enhanced the interaction between Trim32 and DUSP26, while the catalytically inactive PRMT9 (G260E) mutant had no effect on the Trim32‐DUSP26 interaction (Figure 8D). In contrast, PRMT9 knockdown impaired the binding of Trim32 and DUSP26 (Figure 8E,F and Figure S22B). Notably, the binding between Trim32 and DUSP26 R29K mutant was impaired compared to WT DUSP26 protein (Figure S22C). Additionally, we assessed the impact of Trim32 overexpression on the ubiquitination status of DUSP26. The results demonstrated that Trim32 overexpression significantly promoted DUSP26 ubiquitination and this effect was enhanced by co‐expression of PRMT9 (Figure 8G). Conversely, PRMT9 knockdown diminished the stimulatory effect of Trim32 on DUSP26 ubiquitination (Figure 8H), mirroring the effect observed with the DUSP26 R29K mutant (Figure S22D). To investigate whether the degradation of DUSP26 mediated by PRMT9 relied on Trim32, we transfected siRNA specific to human Trim32 into SH‐SY5Y cells and found that Trim32 knockdown mitigated the degradation of DUSP26 induced by PRMT9 overexpression (Figure 8I). Consistently, Trim32 knockdown abolished the effect of PRMT9 on DUSP26 ubiquitination (Figure 8J). Collectively, these results indicated that PRMT9 facilitated Trim32‐mediated ubiquitination and degradation of DUSP26 by arginine methylation at R29.

## 3 Discussion

PD is a progressively neurodegenerative disease derived from the interplay between genes and environmental factors, and insights into the pathogenesis of PD remain to be elucidated. In this study, we found that PRMT9 aggravated MPP^+^‐induced mitochondrial dysfunction in SH‐SY5Y cells. Furthermore, we provided evidence that PRMT9 deficiency alleviated DA neurodegeneration in MPTP‐induced PD model. Additionally, the MPTP treatment upregulated the protein level of PRMT9, which triggered the impairment of DUSP26 and exacerbated the neurodegeneration. Mechanistically, we demonstrated that PRMT9 interacted with DUSP26 directly and negatively regulated DUSP26 by promoting its ubiquitous degradation.

Arginine methylation was widely involved in various neurological disorders, whereas its relation to PD was little known. Like PRMT7, PRMT9 is characterized by a unique duplicated methyltransferase domain [43]. Previous studies revealed PRMT9 was highly expressed in several types of cancer and played a pivotal role in tumorigenesis [16, 44, 45, 46]. The Human Protein Atlas (HPA, https://www.proteinatlas.org/) database showed that PRMT9 was extensively distributed in the nervous system. Alternatively, a recent study demonstrated that PRMT9 G189R mutation was found in intellectual disability indicating its potential function in neurological disorders [17]. However, whether PRMT9 is linked to the development of PD is not clear. The present study showed that PRMT9 deficient mice challenged by MPTP alleviated the behavioral and pathological phenotypes. Consistently, overexpressing PRMT9 specifically in SN further aggravated motor defects in mice subjected to MPTP. Additionally, analysis of the public GEO dataset GSE168496 revealed that PRMT9 mRNA levels were markedly increased in SNpc tissues of PD patients. Further detection of PRMT9 mRNA and protein levels in MPTP‐induced mouse models and MPP^+^‐treated SH‐SY5Y cells confirmed that both mRNA and protein expression of PRMT9 were elevated, suggesting that PRMT9 was upregulated at the transcriptional level in PD. However, the precise upstream molecular mechanisms responsible for PRMT9 upregulation under pathological stress conditions of PD remain to be further explored. Notably, our data confirmed that MPP^+^, a classic Complex I inhibitor specifically toxic to DA neurons, markedly induced PRMT9 upregulation in PD models, and analysis of public databases revealed that PRMT9 expression was unchanged in AD and HD, suggesting that PRMT9 upregulation was probably a PD‐specific event associated with DA neuron degeneration.

As generally accepted, mitochondrial dysfunction plays an essential role in the pathogenesis of both sporadic and familial PD [47]. Accumulated aged and defective mitochondria may release ROS and enhance neuroinflammation, which leads to neuronal death. PRMT9 overexpression indeed aggravated the mitochondrial dysfunction in MPP^+^‐treated SH‐SY5Y cells, which was consistent with the PRMT9 silence results. The data indicated that PRMT9 contributed to the regulation of mitochondrial function and aggravated dopaminergic neurodegeneration in PD model. Our previous studies proved that PRMT9 was located in mitochondria in untreated cells, and then dissociated from the mitochondria after SeV infection [15]. Excitingly, we also observed that MPP^+^ stimulation promoted PRMT9 to translocate into mitochondria, implying that this translocation was likely stress‐induced. A key unresolved mechanistic issue is how PRMT9 accomplishes mitochondrial translocation in the absence of a canonical mitochondrial targeting sequence. It has been reported that multiple OMM proteins, including TOM20, MAVS and USP33, are targeted to mitochondria via a “signal‑anchor” mechanism, defined as transmembrane (TM) domains flanked by positively charged residues [48, 49, 50, 51]. Whether PRMT9 contains a functional TM domain that mediates its mitochondrial localization awaits further experimental validation. In addition, it is also possible that PRMT9's mitochondrial recruitment may occur either by hitching a ride on another protein or through oxidative stress‐induced changes in membrane permeability. Cu/Zn Superoxide Dismutase (SOD1) was imported into the mitochondrial intermembrane space through its oxidation‐dependent interaction with the copper chaperone CCS [52]. Since MPP^+^ strongly induces mitochondrial oxidative stress, an alternative possibility is that PRMT9 may be recruited to mitochondria via an analogous mechanism.

DUSP26, an important protein on OMM, belongs to DUSPs family. Previous studies suggested that DUSP26 was highly expressed in CNS neurons including cortex and midbrain [23]. DUSP26 was shown to protect against ROS‐induced damage to renal tissue through activation of MAPKs [53]. More recently, it was reported that loss of DUSP26 in SH‐SY5Y cells impaired mitochondrial function and induced cell death, and its genetic deletion in mice led to comparable mitochondrial defects and DA neurodegeneration [23]. In agreement with these findings, our study showed an obvious downregulation in DUSP26 protein levels in both SN and STR of MPTP‐induced mice and in SH‐SY5Y cells stimulated by MPP^+^, which negatively correlated with the upregulation of PRMT9. Importantly, the rescue experiments also demonstrated that PRMT9 impaired mitochondrial function in SH‐SY5Y cells exposed to MPP^+^ by regulating DUSP26 stability. Moreover, our observations on p38 activation in PRMT9 overexpressed cells stimulated by MPP^+^ proved that DUSP26‐mediated MAPK signals participated in the regulation of mitochondrial homeostasis [38, 54]. To further confirm the PRMT9 effects on DUSP26, we observed the reduction of DUSP26 and activation of its downstream p38 in PRMT9‐overexpressed SH‐SY5Y cells and mice. Consistently, we also found the increment of DUSP26 and inhibition of its downstream p38 in PRMT9‐knockdown cells or PRMT9 deficiency mice. PRMT9‐mediated arginine methylation markedly reduced DUSP26 levels, leading to sustained p38 phosphorylation. Activated p38 promoted pro‐apoptotic BAX activation and mitochondrial outer membrane permeabilization, triggering cytochrome C release, Caspase‐3 cleavage, and ultimately dopaminergic neuronal apoptosis [55, 56, 57]. Besides, recent studies demonstrated that reversible phosphorylation of mitochondrial proteins was a major mechanism for mitochondrial response to various stimuli and up to 40% of mitochondrial proteins were phosphorylated [58]. We argued another explanation that DUSP26, as a phosphatase, may directly dephosphorylate one or more mitochondrial proteins to regulate mitochondrial function [59]. However, the precise mechanism remains to be clarified.

Protein arginine methylation is an important PTM in cellular processes. At present, little is known about the PTM of DUSP26. DUSP26 contained a non‐catalytic N‐terminal domain and a catalytic C‐terminal domain. Notably, DUSP26 was targeted to the OMM through its N‐terminal targeting sequence [23]. Our results showed PRMT9 downregulated DUSP26 protein levels independent of its transcriptional change. Moreover, we identified the obvious SDMA modification of DUSP26 and proved PRMT9 specially interacted with the N‐terminal domain of DUSP26 and catalyzed its arginine methylation. Notably, as a type II PRMT, PRMT5 is the most widely studied SDMA‐forming enzyme and is involved in multiple physiological and pathological processes [7, 60]. Nevertheless, our results showed that DUSP26 interacted with PRMT9, but not PRMT5, indicating that the regulation of DUSP26 stability was a specific function of PRMT9. This high selectivity makes PRMT9 a promising therapeutic target for PD with potentially fewer side effects.

In addition, our study demonstrated that PRMT9‐mediated SDMA modification of DUSP26 at R29 acted as a specific methyl‐degron that was directly recognized by the E3 ubiquitin ligase Trim32. This conclusion is supported by multiple lines of evidence: (1) the R29K mutation of DUSP26 significantly impaired its interaction with Trim32; (2) the catalytically inactive PRMT9(G260E) mutant retained binding to DUSP26 but failed to enhance the Trim32‐DUSP26 interaction; (3) the R29K mutant completely blocked PRMT9‐induced DUSP26 ubiquitination and degradation. Our findings expanded the functional repertoire of SDMA modifications, and identified a novel methyl‐degron that regulates protein stability in neurodegeneration. It is worth noting that the site‐specific R29 methylation of DUSP26 was only validated in in vitro cellular systems, and direct experimental evidence confirming this modification in MPTP‐lesioned mouse brain tissue is currently lacking. Although the decreased K48‑linked ubiquitination of DUSP26 observed in *Prmt9^CKO^* mice lends indirect support to the in vivo relevance of our proposed pathway, a key limitation remains: definitive in vivo validation of the R29 residue has yet to be performed using site‐specific mutant mouse models.

Previous studies demonstrated that Trim32 suppressed the assembly and activity of mitochondrial Complex I and contributed to heightened mitochondrial dysfunction and facilitated apoptosis of DA neurons in PD models [41, 61]. Intriguingly, this effect aligned with the roles of PRMT9 and DUSP26 in regulating mitochondrial function. Furthermore, in MPP^+^‐stimulated SH‐SY5Y cells, PRMT9 displayed enhanced translocation to mitochondria and a stronger interaction with DUSP26. Notably, following MPP^+^ stimulation, Trim32 also translocated to the mitochondrial outer membrane, exhibiting increased localization [41]. Together, these findings support the formation of a mitochondrial signaling hub in which PRMT9, DUSP26, and Trim32 specifically converge under pathological stress.

In summary, the present study suggested that PRMT9 negatively regulated DUSP26 protein stability through the ubiquitin‐proteasome pathway, which played a critical role in DA neurodegeneration. Our study also demonstrated a novel mechanism of mitochondrial function regulation mediated by arginine methylation. Importantly, PRMT9 would be a novel therapeutic target and pharmaceutical intervention in the interaction between PRMT9 and DUSP26 may provide a promising therapeutic strategy for PD.

## 4 Methods

### 4.1 Animals

C57BL/6 wild‐type (WT) (male, 8–10 W, 23 ± 3 g, animal approval number: SCXK (Jing) 2019‐0010) mice were purchased from SPF (Beijing) Biotechnology Ltd. The *Prmt9^flox/flox^* mice were obtained from Professor Chengjiang Gao. *Prmt9^flox/flox^* mice were crossed with *DAT‐Cre* transgenic mice to generate *Prmt9^flox/flox^/Cre^+^* (*Prmt9^CKO^*) mice. Littermate *Prmt9^flox/flox^/Cre^−^* mice were used as wild type controls. The Genotyping of *Prmt9^flox/flox^* was confirmed by means of PCR using the following primers, forward primer 5’‐GGTTCTCTTCTTCCATCAAGTAG‐3’, reverse primer 5’‐TCTTCCTTAAATACTTCCTCCGTG‐3. The primers for DAT‐Cre transgenic mice genotyping were forward primer 5’‐TGGCTGTTGGTGTAAAGTGG‐3’ and reverse primer 5’‐CCAAAAGACGGCAATATGGT‐3’. All experimental procedures were approved by the Ethics Committee of Shandong University (Ethical approval number: ECSBMSSDU2024‐2‐23).

### 4.2 Brain Adeno‐associated Virus Delivery

Adeno‐associated virus serotype 9 (AAV9) vectors (AAV9‐hSyn‐P2A‐GFP) constructed by WZ Biosciences Inc were used as the negative control. The experimental vector was AAV9‐hSyn‐PRMT9‐3×Flag‐P2A‐GFP, in which the mouse *Prmt9* coding sequence with a C‐terminal 3×Flag tag was driven by the neuron‐specific human Synapsin I (hSyn) promoter. PRMT9‐3×Flag and GFP were co‐expressed in a non‐fused manner via the P2A self‐cleaving peptide. WT mice were anesthetized and placed on a stereotaxic apparatus for bilateral substantia nigra injection of 2 µl of AAV‐PRMT9 or control AAV suspension (1.0 × 10^13^ vg ml^−1^) at a rate of 0.4 µl min^−1^. (coordinates: Bregma ML ±1.3 mm, AP ‐3.0 mm, DV ‐4.7 mm). After the behavioral tests and sample collection, all mice were subjected to post‐hoc verification of injection sites by GFP expression in the SNpc. Mice with off‐target viral injection were excluded from the final analysis.

### 4.3 MPTP‐induced PD Mice Models and Behavioral Measurements

MPTP‐induced PD mice models and behavioral measurements were carried out as previously described [62]. Acute MPTP intoxication paradigms were used to generate PD mouse models with 9‐week‐old male *Prmt9^CKO^* mice and their age‐matched WT littermates. Mice were administered intraperitoneally with 20 mg kg^−1^ MPTP free base (M0896, Sigma‐Aldrich, USA) dissolved in 0.9% saline four times a day every 2 h.

For rotarod test, the motor coordination ability of mice was assessed with a rotarod apparatus (Panlab Harvard Apparatus, USA). In training session, mice were placed toward the inside on rotarod device at a constant 10 rpm for 200 sec. In the test session, mice were placed on rotarod device at an accelerating speed (starting from 0 to 45 rpm in 450 sec). The speed and the duration until mice fell onto the base were recorded and only one trial was conducted.

For the pole test, a 60 cm long and 1 cm diameter‐sized wooden pole wrapped with gauze to prevent slipping was used. Mice were placed head down on the top of the pole and pre‐trained three times. The latency for the mice to climb down from the top of the pole to the base was measured. Trials were considered a failure if the mouse jumped or slid down the pole.

All behavioral measurements in the rotarod test and pole test were performed by an experimenter who was completely blinded to the genotype and treatment groups of the mice, to avoid any subjective bias in the data collection.

### 4.4 Immunohistochemistry

Mice were anaesthetized by intraperitoneal injection of 3% pentobarbital sodium according to their body weight and perfused with 1×PBS, followed by 4% paraformaldehyde (PFA). Brains were rapidly removed and maintained in 4% PFA at 4°C overnight. The fixed brain was transferred to 15%, 30%, and 30% sucrose buffer for gradient dehydration, and then frozen in tissue freezing medium for slicing. Brain sections containing the substantia nigra and striatum were made using frozen section technique (Leica, Germany). Obtained brain sections (20 µm) were stored in PBS containing 1% PFA, blocked endogenous peroxidase, perforated with 0.2% Triton X‐100, and then blocked with 10% fetal bovine serum (FBS). After blocking, brain sections were incubated sequentially with TH (Millipore, Germany) and secondary antibodies.

Quantification of TH positive neurons in SN and the density of dopaminergic terminals in STR were performed in a blinded manner according to the protocol described previously [63]. For SN sections, every seventh section from the starting of SN (around 2.8 mm from bregma) was considered for counting of TH neurons. In detail, images were captured at the same exposure time and analyzed by ImageJ. Threshold was selected under Image/Adjust in order to achieve a desired range of intensity values to show a single neuron for each experiment. Once determined, this threshold was applied to all the images in each experiment. The selected area in the signal intensity range of the threshold was measured by the Analyze Particles. For STR sections, the average optical density of the whole STR was measured using the ImageJ software, and the staining signal was calibrated by subtracting the baseline signal of the cortex. The data of average optical density were normalized to the WT mice injected with control group.

### 4.5 Cell Culture and Transfection

HEK 293T cells and SH‐SY5Y cells were purchased from the Cell Center of Chinese Academy of Sciences (Shanghai, China) and maintained in DMEM (Meilunbio, Cat. MA0212, Dalian, China) with 10% FBS. Lipofectamine 2000 (Invitrogen, Cat. 11668019, USA) was utilized to transfect the indicated cells with plasmid or siRNA (Supplemental Table S1 and S2).

### 4.6 Quantitative Real‐time PCR

Total RNA was extracted using RNA‐Quick purification kit (ES Science, Cat. RN001, Shanghai, China) and then reverse‐transcripted into cDNA using Hiscript II Q RT SuperMix for qPCR (Vazyme, Cat. R223‐01, Nanjing, China) with the following procedure: 50°C, 15 min; 80°C, 15 s. The PCR reaction was carried out using SYBR green qPCR mix (CWBio, Cat. CW2624, Taizhou, China) on StepOne Real‐Time PCR System. β‐actin was used as the house‐keeping gene. Protocol for qPCR was as follows: 45°C, 10 min; 95°C, 10 s and 65°C, 45 s for 40 cycles; 95°C, 15 s; 60°C, 1 min; 95°C, 15 s; 60°C, 15 s. Primers were listed in Supplemental Table S3.

### 4.7 Immunofluorescence Staining

The slides of cells or frozen brain sections (10 µm) were double labeled with indicated primary antibodies at 4°C overnight, and then incubated with the secondary antibodies for 2 h at room temperature. DAPI (Beyotime, Cat.C1005, Beijing, China) was used for nuclear labelling. Antibodies and dilution ratio used in immunofluorescence staining were listed in Supplementary Table S4.

### 4.8 TUNEL Staining

TUNEL staining was performed by an in situ cell death detection kit (KeyGen Biotech, Cat. KGA7063, Nanjing, China). The number of TUNEL‐positive cells was counted in 3 non‐overlapping microscopic eyeshots by a blinded person under high‐power magnification (× 200) and displayed as a percentage.

### 4.9 Cell Viability Measurement

Cell viability was determined by using the cell counting kit‐8 (Beyotime, Cat. C0037, Beijing, China).

### 4.10 Measurement of ATP Levels and Mitochondrial Membrane Potential

The ATP levels and mitochondrial membrane potential of SH‐SY5Y cells were measured 24 h after MPP^+^ treatment by using ATP assay kit (Beyotime, S0026, Haimen, China) and Mitochondrial membrane potential assay kit with JC‐1 (Beyotime, C2006, Haimen, China) according to the manufacturer's recommendations.

### 4.11 Measurement of ROS and Apoptosis by Flow Cytometry

Flow cytometry was performed by Reactive Oxygen Species Assay Kit (Applygen, C1300, Beijing, China) or Annexin V‐APC/PI apoptosis detection kit (Vazyme, A214‐02, Nanjing, China) according to the manufacturer's instructions. For intracellular ROS detection, cells were incubated with Dihydroethidium (DHE) for 30 min at 37°C. For cell apoptosis measurement, 5 µl of Annexin V‐APC and 10 µl of PI were added and the cells were gently vortexed and incubated for 10 min at room temperature in the dark. All cells were collected by centrifuging, then washed, resuspended with cold PBS and analyzed immediately by flow cytometry. Note that cells in the Q1‐UR and Q1‐LR quadrants were determined as apoptotic cells.

### 4.12 Cell Mitochondria Isolation

Crude mitochondria were isolated from SH‐SY5Y cells using an enhanced mitochondria isolation kit (Beyotime, Cat. C3602S, Beijing, China) according to the manufacturer's instructions.

### 4.13 Western Blot and Immunoprecipitation (IP)

Western blot analysis was performed as previously described [64]. For immunoprecipitation, cells were lysed with NP40 buffer and placed on the ice for 30 min. Then the cell lysates were centrifuged at 12,000 rpm at 4°C for 10 min and the supernatants were collected and incubated with the indicated antibodies and corresponding IgG controls at 4°C for 4 h. Next, Protein A+G agarose (Santa Cruz, Cat. Sc‐2003, Dallas, TX, USA) was mixed overnight at 4°C under rotation. Finally, the beads were washed and eluted in SDS sample buffer for further analysis. Antibodies and dilution ratio used in Western blot and IP assay were listed in Supplemental Table S4.

### 4.14 LC‐MS/MS Analysis

To identify the arginine methylation sites of DUSP26, immunoprecipitated Myc‑DUSP26 protein was subjected to LC‑MS/MS analysis. Peptide mixtures were separated using an EASY‑nLC 1000 UPLC system coupled with a Q Exactive Plus Orbitrap mass spectrometer (Thermo Fisher Scientific, Waltham, MA, USA). MS/MS spectra were processed and analyzed with Proteome Discoverer 1.3 software for peptide mapping and characterization of methylated arginine residues. The methylation site at Arg29 of DUSP26 was identified and confirmed based on the corresponding mass spectrometric data.

### 4.15 Primary Neuronal Culture

The primary cultured midbrain neurons were prepared as previously described [65]. In brief, sacrificed pregnant mice and collected E13.5 embryos in dishes and wash them by D‐Hanks buffer (Meilunbio, MA0039). Under dissection microscope, dissected ventral mesencephalon from the embryos. Added Trypsin‐EDTA (Gibco Life Technologies, 25200072) to the dissected brain fragments and digested at 37°C for 15 min. Then removed trypsin by centrifugation, added serum containing‐culture medium and performed mechanical cell dissociation with a pipet (10 trituration movements, repeated twice). Cells were plated on 6‐well plates at a density of 1,000,000 cells per well or Poly‐D‐Lysine Hydrobromide (Gibco Life Technologies, A38904‐01)‐coated glass coverslips in 6‐well plates at a density of 600,000 cells per well.

### 4.16 Docking

The protein‐protein docking was conducted using HADDOCK (High Ambiguity Driven Biomolecular Docking) 2.4. Generated structures were clustered based on the fraction of common contacts, and the resulting complexes were ranked using the HADDOCK scoring function. Intermolecular interactions, including hydrogen bonds, salt bridges, and π‐π stacking at the binding interface, were identified and visualized using PyMOL (www.pymol.org).

### 4.17 Mendelian Randomization Analysis

We used eQTL data from brain tissue in the large public data platform GTEx portal (https://gtexportal.org/home/), filtered out genes from the PRMT family, performed data cleaning, and removed linkage disequilibrium. Then, we conducted two‐sample Mendelian randomization analysis using GWAS data for Parkinson's disease from the Psychiatric Genomics Consortium database (https://pgc.unc.edu/), followed by sensitivity testing, to identify causal associations between them. The IVW method was the main method used in this study, because it can summarize Wald ratios from individual SNPs [66]. On the other hand, the robustness of the study results was evaluated through sensitivity analyses. Heterogeneity effects can be accounted for using the random‐effects model of the IVW method [67], while pleiotropy can be assessed using the MR‐Egger method. If the intercept is very close to zero, there is little evidence of horizontal pleiotropy; however, if the intercept deviates substantially from zero, this suggests the presence of horizontal pleiotropic effects among the instrumental variables (IVs) [68].

### 4.18 Statistical Analysis

Data were represented as mean ± standard deviation (SD). All sample sizes (*n*) were clearly indicated for each statistical comparison. Prior to statistical testing, potential outliers were screened via Grubbs' test, and no valid outliers were identified in all datasets. All statistical analyses were performed using GraphPad Prism software (Version 8.0) (GraphPad Software Inc, San Diego, CA, USA). The differences between two experimental groups were analyzed using Student's *t* test. For multiple comparisons, one‐way or two‐way ANOVA with post hoc Tukey's test was used to determine statistical differences. *p* < 0.05 was considered as statistically significant.

## Author Contributions

H.L. and C.G. designed and supervised the study. T.L., X.S., X.G., R.X., Q.J., L.Y., Y.L., Q.F. and Q.X. performed the experiments and analyzed the results. L.C., R.S., and L.K. contributed to experimental design and discussion. T.L. and X.S. wrote the manuscript. H.L., C.G. and L.C. revised and reviewed the final version of this manuscript. All authors read and approved the final manuscript.

## Ethics Statement

All animal experiments were performed according to the principles established for the care and use of laboratory animals by the National Institutes of Health and were approved by the Institutional Animal Care and Use Committee of Shandong University (Ethical approval number: ECSBMSSDU2024‐2‐23).

## Conflicts of Interest

The authors declare no conflicts of interest

## Supporting information




**Supporting File**: advs76033‐sup‐0001‐SuppMat.docx.

## Data Availability

The data that support the findings of this study are available from the corresponding author upon reasonable request.
