# Supplementary material for: PRMT9 Aggravated Dopaminergic Neurodegeneration in Parkinson's Disease Model by Facilitating the Degradation of DUSP26 and Inducing Mitochondrial Dysfunction
Source: Adv Sci (Weinh). 2026 Jun 9;13(48):e76033. doi: 10.1002/advs.76033 (PMC13336452; doi:10.1002/advs.76033)
Supplement: Supplementary file 1 [file ADVS-13-e76033-s001.docx]

Supporting Information

**PRMT9 Aggravated Dopaminergic Neurodegeneration in Parkinson****'s Disease Model by Facilitating the Degradation of DUSP26 and Inducing Mitochondrial Dysfunction**

Tengfei Liu, Xiaomeng Song, Xin Guo, Rui Xiao, Ru Sun, Qiuran Ji, Lu Yu, Yiquan Li, Qingyi Fu, Qidi Xue, Lingjun Kong, Lin Chen^*^, Chengjiang Gao^*^, Huiqing Liu^*^

**Figure S1 The distribution of PRMT7 in mitochondria after MPP^+^ treatment.** HEK 293T cells were treated with MPP^+^ (300 μM) for 24 h. (**A**, **C**) Quantitative analysis of the colocalization between PRMTs with mitochondria. Pearson's Coefficient was quantified by Image J software. Results were representative of 4 independent experiments. (**B**) Confocal microscopy of HEK 293T cells transfected with plasmids expressing GFP-PRMT9 (green) and DsRed2-Mitored (red). Co-localization was quantified by using Pearson's correlation coefficient method from ImageJ software. Scale bars: 10 μm. Two-tailed Student's *t* test was used for statistical analyses. ***p* < 0.01 compared with indicated group.

**Figure S2 PRMT9 knockdown alleviated MPP^+^-induced mitochondrial dysfunction in SH-SY5Y cells.** SH-SY5Y cells were treated with MPP^+^ (300 μM) for 24 h respectively. Detection of JC-1 signals (**A**) and ATP production (**B**) in SH-SY5Y cells under PRMT9 knockdown. (**C**) Flow Cytometry analysis of ROS production in SH-SY5Y cells transfected with siPRMT9 for 24 h of MPP^+^ treatment. (**F**) Confocal microscopy of mitochondrial fragmentation in SH-SY5Y cells transfected with siPRMT9 for 24 h of MPP^+^ treatment. Results were representative of 4 independent experiments. All data are shown as mean ± SD. Multiple comparisons were evaluated by two-way ANOVA followed by Tukey's test, **p* < 0.05, ***p* < 0.01, ****p* < 0.001 compared with indicated group.

**Figure S3 PRMT9 silence alleviated MPP^+^-induced apoptosis in SH-SY5Y cells.** SH-SY5Y cells transfected with siPRMT9 were subjected to MPP^+^ (300 μM) for 24 h. (**A**) Cell viability was determined by CCK-8 assay. Cell death rate was measured by flow cytometry analysis (**B**) and TUNEL staining (**C**). The protein levels of TH (**D**), Bcl-2 and BAX (**E**), Caspase 3 and Cleaved caspase 3 (**F**) were assessed. Results were representative of 3 or 4 independent experiments. All data are shown as mean ± SD. Multiple comparisons were evaluated by two-way ANOVA followed by Tukey's test, **p* < 0.05, ***p* < 0.01, ****p* < 0.001 compared with indicated group.

 **Figure S4 PRMT9 conditional deﬁciency ameliorated dopaminergic degeneration induced by MPTP.** Western blot analysis of lysates from SN (**A**) or STR (**B-D**) of WT or *Prmt9^CKO^* mice treated with MPTP. The protein levels of PRMT9 (**A**), TH (**B**), Cleaved caspase 3, Bcl-2 and Bax (**C**) were assessed (*n* = 6 mice per group). All data were shown as mean ± SD. Multiple comparisons were evaluated by two-way ANOVA followed by Tukey's test, **p* < 0.05, ***p* < 0.01 compared with indicated group.

**Figure S5 Overexpression of PRMT9 aggravated MPTP-induced dopaminergic degeneration.** Western blot analysis of lysates from STR of WT or PRMT9 overexpressed mice treated with MPTP. The protein levels of TH were assessed (*n* = 6 mice per group). All data were shown as mean ± SD. Multiple comparisons were evaluated by two-way ANOVA followed by Tukey's test, **p* < 0.05, ***p* < 0.01 compared with indicated group.

**Figure S6** **MR analysis of the association between PRMT9 expression and PD risk.** (**A**) Study workflow and Manhattan plot showing MR-based identification of PRMT9 as a candidate PD risk gene, using brain cis-eQTLs as instrumental variables and PD GWAS summary data as outcomes. (**B**) Primary MR results. (**C**) Sensitivity tests showed no significant heterogeneity or horizontal pleiotropy. (**D**) Leave-one-out sensitivity analysis confirmed that the MR estimate was not driven by any single instrumental SNP. (**E**) MR scatter plot showed consistent causal effect estimates across five MR methods.

**Figure S7 The protein levels of PRMT9 were upregulated in PD models induced by MPTP.** (**A**) The protein levels of PRMT9 were assessed in primary cultured neurons subjected to MPP^+^. *n* = 3 independent experiments. (**B**) The protein levels of TH, PRMT9 and DUSP26 in STR were assessed in WT mice injected with 20mg kg^-1^ MPTP 4 times a day, with a 2 h interval, for duration of 7 days. (**C**) Pearson correlation analyses showing the correlations between PRMT9 and DUSP26 expression in STR of WT mice subjected to MPTP. *n* = 6 mice per group. All data were expressed as the mean ± SD. Two-tailed Student's *t* test was used for statistical analyses. ***p* < 0.01 compared with indicated group.

**Figure S8 The protein level of PRMT9 was upregulated in SN of mice induced by α-Syn PFFs.** (**A**) The protein levels of PRMT9 were assessed in SH-SY5Y cells subjected to α-Syn PFFs. *n* = 3 independent experiments. (**B**) Immunoblot analysis of protein expression levels in SN from WT mice at 3 months after α-Syn PFFs injection. *n* = 6 mice per group. All data were expressed as the mean ± SD. Two-tailed Student's *t* test was used for statistical analyses. ***p* < 0.01 compared with indicated group.

**Figure S9 PRMT9 mRNA expression in disease models and human datasets.** (**A**, **B**) Relative PRMT9 mRNA levels in MPP⁺-treated SH-SY5Y cells (*n* = 4 independent experiments) and MPTP-induced PD mouse models (*n* = 6 mice per group). (**C**-**E**) PRMT9 expression profiles in human patient datasets. All data were expressed as the mean ± SD. Two-tailed Student's *t* test was used for statistical analyses. **p* < 0.05 compared with indicated group.

**Figure S10 DUSP26 mRNA expression in human datasets.** DUSP26 expression profiles in PD patients. Data was expressed as the mean ± SD. Two-tailed Student's *t* test was used for statistical analyses. ***p* < 0.01 compared with indicated group.

 **Figure S11 Time-dependent changes in PRMT9 and DUSP26 protein expression following MPP⁺ treatment.** Western blot analysis on the expression of PRMT9 and DUSP26 in SH-SY5Y cells. *n* = 3 independent experiments. All data were expressed as the mean ± SD. One-way ANOVA was used for statistical analyses. **p* < 0.05, ***p* < 0.01, ****p* < 0.001 compared with indicated group.

**Figure S12 PRMT9 downregulated the protein levels of DUSP26.** Western blot analysis on the expression of DUSP26 in lysates of HEK 293T cells after gradient transfection of GFP-PRMT9 plasmid (**A**) or siPRMT9 (**B**). *n* = 3 independent experiments. All data were expressed as the mean ± SD. One-way ANOVA was used for statistical analyses. ***p* < 0.01, ****p* < 0.001 compared with indicated group.

 **Figure S13 PRMT9 did not affect the transcription levels of DUSP26.** (**A**, **B**) Relative DUSP26 mRNA levels in SH-SY5Y cells transfected with GFP-PRMT9 plasmid or shPRMT9. *n* = 3 independent experiments. (**C**, **D**) Relative DUSP26 mRNA levels in HEK 293T cells transfected with GFP-PRMT9 plasmid or siPRMT9. *n* = 3 independent experiments. All data were expressed as the mean ± SD. Two-tailed Student's *t* test was used for statistical analyses. **p* < 0.05 compared with indicated group.

**Figure S14 PRMT9 regulated DUSP26-p38 pathway in PD cell models.** SH-SY5Y cells transfected with Flag-PRMT9 or siPRMT9 were subjected to MPP^+^ (300 μM) for 24 h. (**A**, **B**) The protein levels of DUSP26, p-p38 and p38 were assessed (*n* = 4 independent experiments). All data were shown as mean ± SD. Multiple comparisons were evaluated by two-way ANOVA followed by Tukey's test, **p* < 0.05, ****p* < 0.001 compared with indicated group.

**Figure S15 PRMT9 regulated DUSP26-p38 pathway in PD mouse models.** Western blot analysis of lysates from SN of control or PD mice. (**A**, **B**) The protein levels of DUSP26, p-p38 and p38 were assessed (*n* = 6 mice per group). All data were shown as mean ± SD. Multiple comparisons were evaluated by two-way ANOVA followed by Tukey's test, **p* < 0.05, ***p* < 0.01 compared with indicated group.

**Figure S16 PRMT9 was involved in PD by regulating DUSP26 stability.** In rescue experiments, Flag-PRMT9 and Myc-DUSP26 plasmids were co-transfected into SH-SY5Y cells upon 300 μM MPP^+^ stimulation. (**A**) Cell viability was determined by CCK-8 assay. Cell apoptosis was measured by TUNEL staining (**B**) and flow cytometry analysis (**C**). Results were representative of 4 independent experiments. One-way ANOVA was used for statistical analyses. ***p* < 0.01, ****p* < 0.001 compared with indicated group.

**Figure S17 Colocalization of PRMT9 and DUSP26 in** **primary midbrain neurons upon MPP⁺ stimulation.** Confocal analysis of the co-localization of endogenous PRMT9 (red) and DUSP26 (green) in primary midbrain neurons stimulated by MPP^+^ (300 μM) treatment. Scale bar, 10 μm.

**Figure S18 PRMT9 interacted with and** **catalyzed DUSP26 arginine methylation.**

(**A**) Co-IP analysis of the interaction between recombinant protein Flag-PRMT9 and Myc-DUSP26 incubated in vitro. (**B**) IP analysis of arginine methylation types (ADMA, SDMA, MMA) of DUSP26 in HEK 293T cells transfected with plasmids expressing Myc-DUSP26. (**C**) Co-IP analysis of the exogenous interaction of DUSP26 with PRMT9 or PRMT5. HEK 293T cells were transfected with plasmids expressing Myc-DUSP26 and GFP-PRMT9 or GFP-PRMT5.

**Figure S19 The arginine methylation sites of DUSP26.** (**A**) The identified arginine methylation sites of DUSP26 by LC-MS examination. (**B**) The prediction of arginine methylation sites by methyl-group specific predictor software. (**C**) The amino acid sequence containing R29 in different mammals was conserved.

**Figure S20 PRMT9 promoted the degradation of DUSP26 by catalyzing its arginine methylation.** (**A**) Co-IP analysis of the exogenous interaction of PRMT9 (G260E) mutant with DUSP26 in HEK 293T cells transfected with plasmids expressing GFP-PRMT9 (G260E) and Myc-DUSP26. (**B**) HEK 293T cells were transfected with GFP-PRMT9 expression plasmid and Myc-DUSP26 (WT or R29K mutant). The transfected cells were cultured for 36 h before being incubated with CHX for the indicated time. The levels of Myc-DUSP26 in cells lysates at different time points were detected by western blot analysis. *n* = 3 independent experiments. All data was expressed as the mean ± SD. Two-tailed Student's *t* test was used for statistical analyses. **p* < 0.05 compared with indicated group.

**Figure S21 LTN1 did not affect DUSP26 protein levels.** (**A**) The LC/MS results showed the potential interaction between DUSP26 and Trim32 (or LTN1). (**B**) The protein levels of DUSP26 were assessed. *n* = 3 independent experiments. Two-tailed Student's *t* test was used for statistical analyses. **p* < 0.05 compared with indicated group.

**Figure S22 Arginine methylation of DUSP26 modulated the binding affinity between Trim32 and DUSP26.** (**A**) Molecular docking using HADDOCK software predicted the interaction between Trim32 and DUSP26. (**B**) Co-IP analysis of the endogenous interaction strength between Trim32 and DUSP26 in SH-SY5Y cells transfected with shPRMT9 or negative control. (**C**) Co-IP analysis of the exogenous interaction strength between Trim32 and DUSP26 in HEK 293T cells transfected with Flag-Trim32 and Myc-DUSP26 plasmids (WT or arginine mutants). (**D**) IP analysis of DUSP26 ubiquitination in HEK 293T cells transfected with Flag-Trim32 and Myc-DUSP26 plasmids (WT or arginine mutants).

Table S1 siRNA or shRNA used in this study

| **siRNA** | | |
| --- | --- | --- |
| Gene | Species | sequence (5’-3’) |
| PRMT9 | Human | GCUUAACAACAUCCCAUAUTT |
| PRMT9 | Human | GGUAUCCAUUUGCCAACAATT |
| Trim32 | Human | GCCGCAAGGAAATTCTCCATT |
| **shRNA** | | |
| PRMT9 | Human | GCUUAACAACAUCCCAUAU |

Table S2 Expression plasmids utilized in this study

| Plasmids | Source | Identifier |
| --- | --- | --- |
| GFP-PRMT9 | Professor Chengjiang Gao (Shandong University, Jinan, China | N/A |
| Flag-PRMT9 |  |  |
| GFP-PRMT9(G260E) |  |  |
| HA-ubiquitin |  |  |
| Myc-DUSP26(WT) | MIAOLING BIOLOGY | N/A |
| Myc-DUSP26(R29K) |  |  |
| Myc-DUSP26(R50K) |  |  |
| Myc-DUSP26(R112K) |  |  |
| Myc-DUSP26(R158K) |  |  |

Table S3 Primers for qRT-PCR

| Gene | Species | Forward primer (5’-3’) | Reverse primer (5’-3’) |
| --- | --- | --- | --- |
| Actin | Human/Mouse | GGAAATCGTGCGTGACATTAA | AGGAAGGAAGGCTGGAAGAG |
| DUSP26 | Human | TGGTAACTGGCTTTGGGCTTCTATG | ATGTTGAACGGTTGGCATCTCCTC |
| PRMT9 | Human/Mouse | AGGACTTCGGCACTGCCTAT | TCCTTCACGTCGTGTTTCAGC |

**Table S4 Antibodies for western blot and immunofluorescence experiments**

| **Antibodies** | **Source** | **Identifier** | **Dilution ratio** |
| --- | --- | --- | --- |
| PRMT9 | abclonal | NA | WB 1:1000  IF 1:200 |
| DUSP26 | Abcam | Cat#ab224407 | WB 1:1000  IF 1:50 |
| DUSP26 | Immunoway | Cat#A7414 | WB 1:1000 |
| Bcl2 | Proteintech | Cat# 12789-1-AP | WB 1:1000 |
| Bax | Proteintech | Cat# 50599-2-Ig | WB 1:2000 |
| Trim32 | Proteintech | Cat# 10326-1-AP | WB 1:1000 |
| Trim32 | Santa Cruz | sc-135588 | IF 1:50 |
| p-p38 | CST | Cat# **#**4511 | WB 1:1000 |
| p38 | Proteintech | Cat#14064-1-AP | WB 1:1000 |
| Tom20 | BD | Cat#612277 | WB 1:2000 |
| TH | Millipore | MA13318 | WB 1:1000  IF 1:200 |
| Cleaved Caspase 3 | Immunoway | Cat# YC0006 | WB 1:1000 |
| Caspase3 | Immunoway | Cat#19677-1-AP | WB 1:1000 |
| GAPDH | Proteintech | Cat# 60004-1-Ig | WB 1:20000 |
| FLAG | Sigma | Cat#F1804 | WB 1:2000  IF 1:200 |
| GFP | Santa Cruz | Cat#sc-9996 | WB 1:1000 |
| 6*His | Proteintech | Cat#66005-1-1g | WB 1:1000 |
| Myc | Origene | TA150121 | WB 1:1000  IF 1:200 |
| ADMA | CST | Cat#13522 | WB 1:1000 |
| SDMA | CST | Cat#13222 | WB 1:1000 |
| MMA | CST | Cat#8015 | WB 1:1000 |
| NeuN | Millipore | Cat#MAB377 | IF 1:200 |
| GFAP | Millipore | Cat#MAB360 | IF 1:200 |
| IBA1 | Proteintech | Cat#10904-1-AP | IF 1:200 |
| HRP-conjugated Affinipure Goat Anti- Mouse IgG | Proteintech | Cat#SA00001-1 | WB 1:5000 |
| Coralite594-conjugated Goat Anti-Mouse IgG(H+L) | Proteintech | Cat#SA00013-3 | IF 1:200 |
| HRP-conjugated Affinipure Goat Anti- Rabbit IgG | Proteintech | Cat#SA00001-2 | WB 1:5000 |
| Coralite488-conjugated Goat Anti-Rabbit IgG(H+L) | Proteintech | Cat#SA00013-2 | IF 1:200 |
